# Trust and Perceived Usefulness in GenAI-Assisted Assessment Adoption Among Design Teachers in Higher Education: Distinct Judgments and Their Associations with Behavioral Intention

**DOI:** 10.3390/bs16101930

**Published:** 2026-10-17

**Authors:** Jie Wang, Yuri Sim

**Affiliations:** Department of Smart Experience Design, Graduate School of Techno Design, Kookmin University, Seoul 02707, Republic of Korea

**Keywords:** generative artificial intelligence, assisted assessment, trust, perceived usefulness, behavioral intention

## Abstract

Generative artificial intelligence (GenAI) is increasingly being considered for assessment-related work, but educators need to judge whether it is reliable enough to serve as professional input and whether it is useful enough to justify its incorporation into assessment practice. This study examined whether trust (TR) and perceived usefulness (PU) are empirically distinct judgments and whether their independent associations with behavioral intention (BI) to use GenAI for assessment differ. Cross-sectional survey data from 352 design teachers at universities and higher vocational colleges in China were analyzed using confirmatory factor analysis, covariance-based structural equation modeling, comparisons of alternative structural specifications, and 5000-sample bootstrap tests. Measurement evidence supported treating TR and PU as separate latent judgments. In the correlated parallel model (Model C2), PU was positively associated with BI, whereas the TR–BI association was nonsignificant; a formal test of the unstandardized path coefficients showed that the PU–BI coefficient was significantly larger than the TR–BI coefficient. Because Model C2 was structurally just-identified, its global fit was not interpreted as independent evidence of structural robustness. Sensitivity analyses accounting for prior GenAI-assisted assessment use yielded the same substantive path pattern. The findings concern acceptance judgments and behavioral intention rather than actual adoption or assessment outcomes. They suggest that reliability and perceived task value are related but nonredundant considerations when educators consider GenAI support while retaining final assessment responsibility.

## 1. Introduction

Generative artificial intelligence (GenAI) has rapidly expanded the range of tasks that can be supported in educational assessment, including organizing assessment criteria, summarizing student performance, drafting feedback, and generating preliminary assessment suggestions. Because these uses remain embedded in educator-led assessment, explainability, human oversight, data governance, and clear lines of responsibility are central to their appropriate use (Bower et al., 2024; Kizilcec et al., 2024; Miao & Holmes, 2023; Nguyen et al., 2025; Xia et al., 2024). Evidence from second-language faculty in Chinese higher education suggests that GenAI is more readily used for formative support than for high-stakes summative assessment (Iqbal et al., 2025). In summative contexts, educators remain particularly concerned about system explainability and trust, as well as the preservation of professional and contextual knowledge in assessment judgments (van den Berg & Papadopoulos, 2025).

These features make assessment a consequential context in which technology-acceptance judgments are formed under retained professional responsibility. An educator may already use GenAI to prepare lessons, revise materials, or perform routine text-based tasks while remaining reluctant to incorporate it into assessment processes that directly affect students’ academic outcomes. Recent Behavioral Sciences studies have shown that performance expectancy or perceived usefulness, technology self-efficacy, social influence, and related acceptance beliefs can be associated with educators’ or pre-service teachers’ behavioral intentions toward GenAI (Wang et al., 2024; Y. Liu et al., 2025; Hao et al., 2026). The unresolved issue is therefore not simply whether favorable beliefs are associated with intention, but whether educators make two related yet distinct judgments: whether they trust (TR) a system and its outputs sufficiently to use them as professional input and whether they perceive the technology as sufficiently useful (PU) to justify its incorporation into assessment practice. Previous research has examined factors shaping university teachers’ adoption of GenAI assessment tools (Khlaif et al., 2024), but direct evidence remains limited regarding whether TR and PU are empirically distinguishable and whether their independent associations with behavioral intention (BI) differ when educators retain final responsibility for assessment. The present study therefore shifts the focus from identifying additional antecedents of GenAI adoption to clarifying the distinct roles of TR and PU in acceptance judgments related to the intention to use GenAI for assessment.

### 1.1. Teacher Responsibility and Acceptance Judgments in GenAI-Assisted Assessment

In this study, GenAI-assisted assessment refers to the use of GenAI to provide working materials, such as formulations of assessment criteria, summaries of student performance, draft feedback, or preliminary suggestions, while educators retain the authority to review, revise, reject, and make final decisions. The term “assisted” does not constitute a formal disclaimer of responsibility. Even fluently generated text can omit task conditions, misinterpret student performance, or apply generic advice to a mismatched course context. Educators therefore need to evaluate GenAI outputs against course objectives, assessment criteria, and concrete evidence; content that cannot be verified or is clearly unsuitable should not be incorporated directly into formal assessment (Bower et al., 2024; Nguyen et al., 2025; Xia et al., 2024).

Using GenAI to organize materials and allowing it to contribute to feedback or grading do not entail equivalent levels of risk. The latter involves greater verification demands, interpretive responsibility, and potential consequences of error. Accordingly, relevant considerations include not only whether outputs are sufficiently consistent and whether their underlying basis is understandable, but also whether the effort required for verification offsets the anticipated efficiency gains and how responsibility will be allocated when problems arise. Performance expectancy, perceived usefulness, self-efficacy, training, and institutional support have been identified as important conditions shaping teachers’ adoption of GenAI (Hazzan-Bishara et al., 2025; Khlaif et al., 2024). At the same time, assessment research and international guidance repeatedly emphasize academic integrity, human oversight, data use, and clear boundaries of accountability (Miao & Holmes, 2023; Nguyen et al., 2025; Xia et al., 2024). Thus, technical availability does not automatically translate into educators’ willingness to incorporate GenAI into assessment practice.

Design education makes this tension particularly visible. Studio assessment rarely involves determining a single correct answer; instead, educators typically integrate evidence concerning the final product, learning process, student engagement, and design quality into an overall judgment. Although predetermined criteria can provide reference points, they cannot eliminate uncertainty in complex assessment tasks (Sadler, 2009). Art and design educators interpret relationships among students’ work, processes, and meanings (Orr & Bloxham, 2013), while educators and students may differ in how they understand the usability, design, and assessment functions of analytic rubrics (Abdellatif & El-Wakeel, 2025). Research on creativity in design education likewise indicates that assessment involves multiple dimensions, including novelty, elaboration, and the organization of design elements (Demirkan & Afacan, 2012). These characteristics motivate treating GenAI as support for, rather than a substitute for, educators’ integrated assessment judgments concerning evidence, meaning, and context.

Design education is therefore treated in this study as a context in which professional judgment and responsibility are particularly salient. Because the questionnaire did not directly measure visual evidence, creative processes, aesthetic judgment, or design intent, and because no comparison group from other disciplines was included, the study does not claim a design-specific mechanism. Instead, design education defines the empirical context and sample within which these acceptance judgments were examined; the results are not interpreted as evidence that the observed associations are unique to design educators.

### 1.2. “Trustworthy Enough to Consider” and “Useful Enough to Use”: Two Related but Distinct Acceptance Judgments

TR and PU can both represent positive evaluations of GenAI, but they address different questions. In the present study, TR refers to the judgment that a GenAI system and its outputs are sufficiently stable and reliable to serve as professional input under human oversight. PU refers to the judgment that GenAI can improve assessment efficiency, enhance assessment quality, and support specific assessment tasks. These constructs draw on established perspectives on TR in automation and technology acceptance, which distinguish judgments about system reliability from judgments about the benefits that technology provides (Davis, 1989; Glikson & Woolley, 2020; Lee & See, 2004; McGrath et al., 2025).

The two judgments may be positively related because perceptions of reliable system performance can contribute to favorable evaluations of its usefulness. However, they are not interchangeable. A tool may offer meaningful efficiency gains while remaining insufficiently reliable for professional use, or it may produce consistent outputs without reducing the effort required for verification, interpretation, and organization. In assessment contexts, this distinction is particularly consequential because educators may consider anticipated usefulness alongside the additional work and responsibility associated with checking and integrating GenAI outputs. Recent research with higher-education instructors similarly suggests that willingness to consult certain GenAI outputs can coexist with concerns about errors, misleading information, or skill degradation (Lyu et al., 2025). Thus, educators may regard GenAI outputs as sufficiently trustworthy to consider while still judging the technology insufficiently useful to warrant routine incorporation into assessment practice.

Existing studies have identified PU, TR, training, and institutional support as important factors associated with GenAI adoption, but direct evidence remains limited regarding whether TR and PU have equivalent independent associations with BI when modeled simultaneously (Ayanwale et al., 2024; Hazzan-Bishara et al., 2025; Khlaif et al., 2024; Viberg et al., 2025). Recent Behavioral Sciences studies likewise embed usefulness/performance expectancy, self-efficacy, trust, and BI within broader GenAI adoption frameworks (Wang et al., 2024; Y. Liu et al., 2025; Zhang & Hu, 2025; Hao et al., 2026), but they do not directly test whether TR and PU are empirically distinct latent judgments or whether their unique associations with BI differ significantly. Existing models also differ in where they position the two constructs. Some specify a TR → PU relationship, others treat TR as a conditioning factor, and still others position perceived benefits as antecedents of TR (Choung et al., 2023; Kılıç & Çelik, 2025; Shata & Hartley, 2025). These alternative formulations indicate that the structural relationship between TR, PU, and BI remains theoretically unsettled. The present study therefore addresses three related questions: whether the measurement evidence supports treating TR and PU as separate latent judgments, whether their independent associations with BI differ in magnitude, and how the interpretation of the TR–PU–BI relations changes across alternative structural specifications.

AI self-efficacy (AISE) in the assessment context represents a capability-related belief. Educators who believe they can configure tasks, interpret outputs, and respond appropriately to anomalous results may be better positioned to evaluate system reliability and practical value. Recent Behavioral Sciences research likewise treats GenAI-related self-efficacy as a psychologically meaningful belief related to readiness among teacher populations (Wang et al., 2024; X. Liu et al., 2026). In the present study, however, AISE remains a subjective belief in one’s capabilities rather than an objective measure of AI literacy or verification performance. Accordingly, AISE is retained as an important component of the model but is not the primary theoretical focus; it provides a capability-related basis for examining TR and PU in the focal model.

### 1.3. Research Questions and Analytical Strategy

These considerations lead to three research questions:

RQ1. Does the measurement evidence support treating trust (TR) and perceived usefulness (PU) as separate latent judgments in the context of GenAI-assisted assessment?
RQ2. When TR and PU are modeled simultaneously, do their independent associations with BI differ significantly?
RQ3. When TR and PU are specified in different directional or correlated-parallel structures, how sensitive is the interpretation of the TR–PU–BI relations to model specification?

The analysis begins with measurement distinctiveness because comparing the roles of TR and PU is only meaningful if the measures support treating them as separate latent judgments. This comparison provides the measurement basis for RQ2 and is treated as a prerequisite for the structural analysis rather than as the study’s primary substantive contribution.

To address RQ3, the study compares focal-direction, reverse-direction, independent-parallel, and correlated-parallel structures using the same measurement basis to assess specification sensitivity. The formal TR–BI versus PU–BI comparison is conducted within Model C2, whereas the alternative structures are used to examine how interpretation depends on structural specification, not as independent replications of the coefficient-difference result.

The study’s incremental contribution lies in combining measurement differentiation with a formal within-Model-C2 path-difference test while using alternative structural specifications to delimit interpretation of the TR–PU relationship. The resulting claims concern acceptance judgments and intention rather than observed assessment performance.

## 2. Theoretical Framework and Hypotheses

The framework clarifies the distinct function of each construct in teacher-supervised, GenAI-assisted assessment before specifying a focal structure alongside alternative structural specifications.

### 2.1. GenAI-Assisted Assessment as Professional Judgment Support Under Teacher Supervision

This study conceptualizes GenAI-assisted assessment as a form of professional judgment support provided under teacher supervision. GenAI may formulate assessment criteria, summarize evidence, draft feedback, or generate preliminary suggestions, but these outputs remain working materials subject to educator review, revision, rejection, and final decision-making (Bower et al., 2024; Miao & Holmes, 2023; Nguyen et al., 2025; Xia et al., 2024). When assessment decisions may affect students’ interests, potential efficiency and personalized support should be considered alongside explainability, contextual knowledge, and accountability. This arrangement is consistent with a human-in-the-loop logic in which AI supports evidence organization and preliminary judgment while educators retain interpretive authority, verification responsibility, and final decision-making power (van den Berg & Papadopoulos, 2025).

Accordingly, the empirical outcome of this study is BI—educators’ intention to use GenAI in assessment-related work—rather than actual adoption, sustained use, or assessment effectiveness. References to “adoption” in the theoretical literature are therefore treated as background to technology-acceptance judgments rather than as an observed outcome in the present data. Design education provides a setting in which open-ended professional judgment is particularly salient, while its design-specific features are treated as contextual rather than as explanations for the observed statistical associations.

### 2.2. Assessment-Context AI Self-Efficacy as a Capability Basis for Engaging with AI-Assisted Assessment

Self-efficacy concerns an individual’s judgment of their capability to organize and execute a particular course of action, and such judgments are specific to the task or context in which the capability is exercised (Bandura, 1982). Recognizing the educational potential of GenAI does not necessarily mean that an educator feels capable of using it for assessment. An educator may be comfortable with general digital tools while remaining uncertain about how to configure an assessment task, interpret generated output, or respond when suggestions conflict with assessment criteria and available evidence.

Assessment-context AISE is defined here as educators’ belief in their capacity to use GenAI to perform assessment-related tasks, including supporting student reflection and self-assessment, designing or improving assessment approaches, and analyzing student performance to generate useful assessment input. The construct retains the subjective nature of self-efficacy while being specifically contextualized to assessment-related tasks (Xia et al., 2026).

In the focal working model (Model A), AISE is specified as positively associated with TR. Educators who feel more capable of operating and understanding the system are better positioned to determine whether its outputs are consistent, usable as a reference, and appropriate for the task. Recent research also links self-efficacy, institutional support, and professional development to teachers’ GenAI use and TR judgments (Hazzan-Bishara et al., 2025; Nazaretsky et al., 2022; Viberg et al., 2025).

Accordingly, we propose:

**H1.**  *Assessment-context AISE is positively associated with TR in GenAI-assisted assessment among design teachers in higher education.*

AISE may also remain related to BI beyond TR and PU. Even when educators judge the system as reliable and useful, BI may remain limited if they do not believe they can initiate use, identify anomalous outputs, or integrate the tool into existing workflows. The model therefore retains the AISE → BI path to test whether capability beliefs provide an independent basis for behavioral intention.

**H2.**  *Assessment-context AISE is positively associated with BI to use GenAI-assisted assessment among design teachers in higher education.*

### 2.3. Trust and Perceived Usefulness as Related but Non-Interchangeable Judgments

TR concerns the extent to which educators are willing to treat a system and its outputs as reliable enough to serve as professional input. The items in this study address overall trustworthiness, operational stability, and whether generated results can serve as a reference for assessment judgment. This definition follows the trust-in-automation literature on reliability, dependability, and appropriate reliance (Jian et al., 2000; Lee & See, 2004) and is consistent with McGrath et al.’s (2025) validation of a short-form automation trust scale in AI contexts.

PU concerns whether the technology provides practical value for assessment work. Following Davis (1989), the present study examines whether educators perceive GenAI as improving assessment efficiency, enhancing assessment quality, and helping them complete assessment tasks. It therefore measures perceived work-related benefits rather than a general attitude toward AI or its future potential. An educator may recognize that GenAI speeds up feedback organization while still doubting its accuracy; conversely, a system may appear stable without reducing workload or improving assessment quality. Because such mismatches can occur in practice, evaluating whether the measurement evidence supports treating TR and PU separately is a prerequisite for analyzing their different roles.

Although TR and PU are not interchangeable, they may still be positively related. For assessment tools that require educator review, reliability judgments may be considered alongside anticipated checking and revision demands when educators evaluate practical value. Judgments of dependability may therefore co-occur with higher evaluations of work value. Meta-analytic work integrating TR into technology acceptance models and recent AI adoption studies indicate a relationship between TR and PU, although its direction and position vary across technologies and model specifications (Choung et al., 2023; Kılıç & Çelik, 2025; Shata & Hartley, 2025; Wu et al., 2011). Accordingly, H3 tests whether TR and PU are positively associated, while Model A specifies TR → PU as the focal structural relationship. Because the data are cross-sectional, this specification represents a theoretically proposed association rather than evidence of a temporal causal sequence. Alternative structural specifications examine whether the association pattern changes when the direction of the TR–PU relationship is reversed or the constructs are modeled in parallel. The corresponding hypothesis is:

**H3.**  *TR is positively associated with PU among design teachers in higher education.*

### 2.4. Independent Relations Between Two Judgments and Behavioral Intention

PU directly addresses whether GenAI provides sufficient practical value to warrant incorporation into the assessment workflow. Educators’ willingness to use GenAI in assessment is expected to be related to perceived task-level benefits, including whether it is seen as reducing repetitive organizational work, improving perceived assessment quality, or helping structure assessment materials. Technology acceptance research and recent studies of teachers’ GenAI adoption consistently identify usefulness, performance expectancy, and concrete work-related benefits as important bases for BI (Davis, 1989; Hazzan-Bishara et al., 2025; Khlaif et al., 2024; Kılıç & Çelik, 2025; Shata & Hartley, 2025).

The independent relationship between TR and BI is less clear. Educators may regard some outputs as worth consulting while remaining concerned about errors, misleading information, or skill degradation (Lyu et al., 2025). In formal assessment, judging an output to be sufficiently trustworthy as a limited reference does not necessarily mean judging GenAI sufficiently valuable to warrant routine incorporation into the assessment workflow. One theoretical possibility, not directly measured in this study, is that expected checking or correction costs may weaken the practical value attributed to otherwise trustworthy outputs.

Thus, RQ2 does not compare the simple correlations of TR and PU. Rather, it asks whether each construct retains an independent association with BI when both are entered simultaneously into the same model. Nor does the study infer a significant difference between coefficients merely because one path is significant and the other is not; establishing that the two associations differ requires a formal path-difference test.

**H4.**  *PU is positively associated with BI for GenAI-assisted assessment among design teachers in higher education.*

### 2.5. Focal Working Model and Alternative Structural Specifications

Bringing these relationships together, this study first estimates a focal directional structure, AISE → TR → PU → BI, while retaining a direct AISE → BI path. This structure treats AISE as a capability basis for engaging with assessment practice, TR as a dependability judgment, and PU as a work-value judgment. It serves as the initial working representation of the proposed relationships rather than as an assumption that educators necessarily form these judgments in a fixed temporal order.

Within this structure, a specific serial indirect association may connect AISE and BI through TR and PU. This test examines whether the corresponding product of paths is statistically supported in Model A; it does not imply that a temporally unfolding mediation process has been observed in cross-sectional data.

**H5.**  *Within the serial structure specified in Model A, assessment-context AISE has a positive specific serial indirect association with BI through TR and PU.*

The TR → PU relationship is not the only plausible structural specification. Prior research does not consistently specify the direction of the relationship between the two constructs: perceived benefits may precede TR, while the two judgments may also operate as parallel evaluations (Ayanwale et al., 2024; Viberg et al., 2025). To avoid treating the focal directional model as the only theoretically plausible specification, these alternatives are represented as alternative structures estimated from the same measurement model.

**Figure 1:**
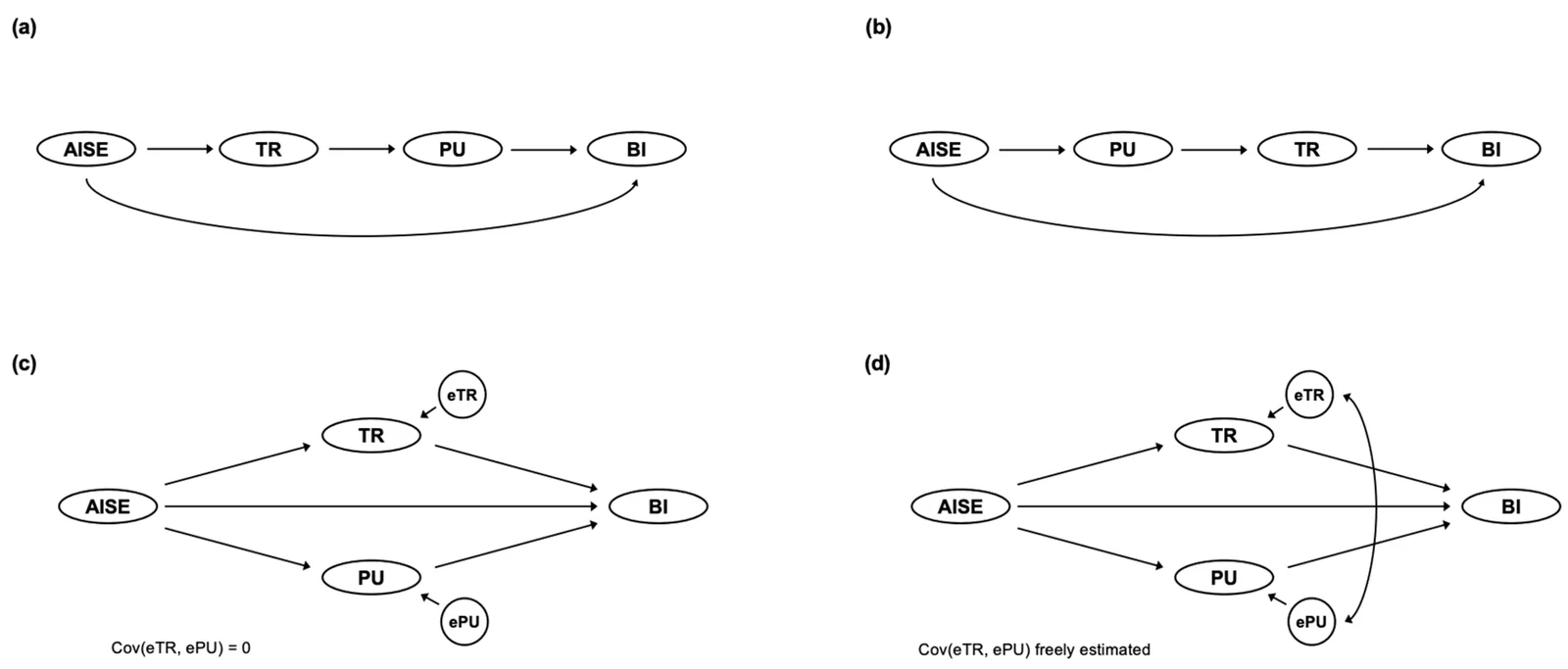
Hypotheses and Alternative Structural Specifications. Note. (**a**) Model A is the focal directional model (TR → PU); (**b**) Model B is the reverse directional model (PU → TR); (**c**) Model C is the strict independent parallel model, with the TR–PU disturbance covariance fixed at 0; (**d**) Model C2 is the correlated parallel model, with the TR–PU disturbance covariance freely estimated. All models retain the direct AISE → BI path. AISE = assessment-context AI self-efficacy; TR = trust in GenAI-assisted assessment; PU = perceived usefulness of GenAI-assisted assessment; BI = behavioral intention to use GenAI-assisted assessment. In panels (**c**,**d**), eTR and ePU denote the disturbance terms of TR and PU, respectively.

Model A specifies TR → PU as the focal directional model. Model B reverses this relation to PU → TR. Model C specifies parallel TR and PU paths with their disturbance covariance fixed at zero, whereas Model C2 retains the parallel paths while allowing the disturbances of TR and PU to correlate. The Extended Model adds AISE → PU and TR → BI to Model A. Model C2 provides the most direct test of RQ2; Model A provides the parsimonious focal working representation; and the remaining models assess whether the core conclusion depends on a particular directional specification or an overly restrictive structural assumption. Figure 1 presents Models A, B, C, and C2; the Extended Model is reported separately as an additional structural specification.

Model comparisons consider absolute fit, information criteria, and structural parsimony. Lower Akaike information criterion (AIC) or Bayesian information criterion (BIC) values indicate that a candidate model summarizes the observed covariance structure at a lower relative cost; they do not establish the correctness of a temporal ordering or causal mechanism. Cross-sectional data and model-selection uncertainty therefore require a clear distinction between a comparatively well-supported structural representation and a confirmed psychological process (Burnham & Anderson, 2004; Maxwell et al., 2011; Preacher & Merkle, 2012).

## 3. Method

### 3.1. Research Design and Context

This study used a cross-sectional survey design and covariance-based structural equation modeling to examine the relationships among assessment-context AISE, TR, PU, and BI. Participants were educators teaching design-related subjects at universities and higher vocational colleges in China, including visual communication, product design, environmental and spatial design, animation and digital media, fashion design, and interaction and service design.

At the beginning of the questionnaire, GenAI-assisted assessment was defined as the use of GenAI to support educators in assessing student assignments, design work, learning processes, or classroom performance. Examples included organizing assessment criteria, analyzing work content, generating feedback suggestions, refining feedback wording, and improving assessment efficiency. The questionnaire also clarified that GenAI functioned solely as an assistive tool and that educators retained responsibility for final assessment judgments.

All four core constructs were measured at the same time point. Accordingly, arrows in the structural models represent theoretically specified directions of association and alternative structural arrangements rather than observed temporal precedence. The analyses therefore do not interpret the estimated paths as evidence of causal relationships or a temporally unfolding psychological mediation process.

### 3.2. Participants, Data Collection, and Participant Protection

Participants were recruited between 2 May and 21 May 2026, through an online survey using purposive nonprobability sampling. Recruitment materials were circulated through university teacher groups, departmental and disciplinary networks, academic communities, and peer referrals. The target population comprised adult educators teaching design-related subjects who had direct experience with assessment activities, including assignment critique, formative feedback, grading, interpretation of assessment criteria, guidance of student self-assessment, or summative assessment. This purposive, network-based recruitment strategy was not intended to produce a probability sample representative of all Chinese design educators.

Experience using GenAI for assessment was not required for participation. Both educators who had previously used GenAI for relevant assessment tasks and those who reported no prior assessment-related GenAI use were eligible, provided that they understood the basic assessment context in which GenAI could be used and were able to evaluate its potential based on their teaching experience. Because the target outcome was prospective BI, prior use was not required; the resulting heterogeneity in experience was addressed explicitly through the use-frequency sensitivity analysis described below.

The survey yielded 400 responses. Sequential screening excluded 12 clearly invalid responses involving missing core items, duplicate records, or invariant response patterns; 15 responses that failed the eligibility criteria; and seven incomplete or low-quality responses based on completion time (<60 s), partial core-item completion, or patterned responding. A further 14 responses were excluded only when Q3 = 2, Q14 = 5, and Q16 = 5 occurred simultaneously; isolated discrepancies did not trigger exclusion. The final analytical sample comprised 352 valid responses (88.0%). Detailed operational criteria and exclusion counts are provided in Supplementary Materials, Section S2 (Table S6). All screening criteria were applied before the confirmatory and structural analyses.

Participant protection procedures were incorporated into the survey design. The opening page described the study purpose, participation procedures, and intended use of the data. Participation was voluntary, and respondents could discontinue the survey before submission. Only respondents who indicated agreement with the study information could proceed to the questionnaire. The questionnaire did not request participants’ names, telephone numbers, email addresses, or specific institutional names. Survey responses were used solely for this study. Demographic characteristics of the final sample are reported in Supplementary Materials, Section S2 (Table S7).

### 3.3. Measures

The four core constructs were measured using five-point Likert scales ranging from 1 (strongly disagree) to 5 (strongly agree). Items were drawn from or informed by established English-language measures. Before forward translation, the research team reviewed the source or source-informed items and established context-adapted English reference wording for the GenAI-assisted assessment setting. The first author then translated the context-adapted items into Chinese for higher-education design teachers, including respondents with and without prior assessment-related GenAI use. ChatGPT-assisted bilingual wording checks (OpenAI; GPT-5.4) were used only for linguistic refinement before the Chinese pilot, with all wording decisions reviewed and confirmed by the researchers. Contextual changes retained the intended content of the source constructs and did not introduce additional dimensions such as visual evidence or aesthetic judgment. Complete construct definitions, final administered Chinese items, and reconciled English versions verified through two independent blinded back-translations are provided in Supplementary Materials, Section S1 (Tables S1–S5). The 13 core measurement items were administered within Question 17 (Q17) of the original Chinese questionnaire; their item IDs are used consistently in the analyses and Supplementary Materials.

Before the formal survey was distributed, 12 higher-education design educators with backgrounds comparable to those of the target sample and spanning multiple design disciplines completed the Chinese pilot questionnaire, which was used to assess scenario clarity, wording, questionnaire flow, possible repetition, and approximate completion time. Following the pilot and subsequent wording refinements, two independent Chinese–English bilingual experts with design backgrounds, neither of whom had participated in the initial translation or contextual adaptation, independently back-translated the candidate Chinese items. Neither expert was given access to the original source items or the predetermined context-adapted English reference wording during back-translation. The research team compared both back-translations with the context-adapted English reference wording and, where necessary, the source-scale wording, focusing on construct meaning, assessment context, and semantic scope. Discrepancies were reviewed and reconciled before the final Chinese version was confirmed. All four AISE items were revised between the pilot and final versions; the nine TR, PU, and BI Chinese items remained unchanged. These procedures assessed comprehensibility and semantic consistency; they were not treated as reliability, validity, or cross-language measurement invariance tests. Detailed records are provided in Supplementary Materials, Section S1.1 (Table S5A–C).

Assessment-context AISE was measured with four items (AISE1–AISE4) primarily informed by the assessment-related content of the GenAI Competency Self-Efficacy Scale for Teachers and Researchers (GAICS-TR), which Xia et al. (2026) developed in the higher-education context. This source was selected because it was developed for higher-education teachers and researchers and contains assessment-related GenAI self-efficacy content closely aligned with the present context. The items captured educators’ perceived ability to use GenAI for assessment-related tasks, including completing assessment-related tasks, supporting student self-assessment, designing or improving assessment approaches, and analyzing student performance to generate useful assessment information.

TR was measured with three items (TR1–TR3) adapted from the three-item short form of the Trust in Automation Scale (S-TIAS) validated by McGrath et al. (2025), selected as a concise measure of AI/automation trust that is conceptually distinct from perceived usefulness. The items focused on educators’ overall TR in GenAI, confidence in its operational stability, and perceptions of whether its generated results could be used as a reference for assessment judgment. PU was measured with three items (PU1–PU3) adapted from Davis (1989), whose perceived-usefulness construct captures perceived work-related benefits of technology use. The items assessed the extent to which educators perceived GenAI as improving assessment efficiency, enhancing assessment quality, and facilitating the completion of assessment work. Finally, BI was measured with three items (BI1–BI3) informed by Venkatesh et al. (2003), whose behavioral-intention measure represents prospective intention to use technology rather than actual use. The items addressed educators’ intention to use or continue using GenAI for assessment and their intention to prioritize GenAI-assisted assessment when conditions permit. Throughout the study, BI specifically refers to intention to use GenAI and is not interpreted as evidence of actual adoption, sustained use, or assessment performance.

### 3.4. Data Screening, Preliminary Analyses, and Common-Method Assessment

SPSS 28.0 was used for data screening, descriptive statistics, reliability analysis, preliminary factor-structure diagnostics, and correlation analyses. Following sample screening, missing data, means, standard deviations, skewness, and kurtosis were examined, together with response times and response patterns, to identify potential anomalies. Internal consistency was assessed using Cronbach’s α and corrected item–total correlations.

The Kaiser–Meyer–Olkin (KMO) statistic, Bartlett’s test of sphericity, principal component extraction, rotated loadings, and scree plot were used before confirmatory analysis as preliminary diagnostics rather than as evidence for the final factor structure. A summary of these preliminary diagnostics is provided in Supplementary Materials, Section S3 (Tables S8–S12 and Figure S1). The distinctiveness of the four constructs was ultimately evaluated through confirmatory factor analysis and comparisons of alternative measurement models.

Because all four core constructs were measured in the same self-report questionnaire, common-method variance was examined in two stages. The preliminary unrotated first-component diagnostic is treated only as a descriptive check. A stronger post hoc CFA-based sensitivity analysis was therefore conducted using a constrained unmeasured latent method factor (ULMF), consistent with general recommendations for post hoc evaluation of common-method variance (Podsakoff et al., 2003; Richardson et al., 2009). All 13 observed items retained their substantive-factor loadings and additionally loaded on an orthogonal common method factor; the unstandardized method-factor loadings were constrained to equality across items, and the method factor was scaled for identification. Model convergence, improper estimates, global fit, substantive standardized loadings, AVE, and CR were compared with the original four-factor measurement model. Because no a priori marker variable was included, the ULMF analysis is interpreted as a sensitivity check rather than as evidence that common-method bias has been eliminated.

### 3.5. Measurement Model, Structural Comparisons, and Bootstrap Procedures

AMOS 27.0 was used for confirmatory factor analysis, structural model comparisons, and bootstrap tests with maximum likelihood estimation. The analyses followed a two-step procedure in which the measurement model was evaluated before structural models were compared (Anderson & Gerbing, 1988). Model fit was assessed using χ^2^ and degrees of freedom, the comparative fit index (CFI), the Tucker–Lewis index (TLI), the root mean square error of approximation (RMSEA), and the standardized root mean square residual (SRMR), with fit indices interpreted in relation to model purpose and comparative fit rather than against isolated cutoff values (Hu & Bentler, 1999).

The measurement analysis first estimated a four-factor model in which AISE, TR, PU, and BI were specified as distinct latent constructs. Standardized factor loadings, composite reliability (CR), and average variance extracted (AVE) were used to assess convergent validity, while the Fornell–Larcker criterion and heterotrait–monotrait ratio (HTMT) were used to assess discriminant validity (Fornell & Larcker, 1981; Henseler et al., 2015; Rönkkö & Cho, 2022). To address RQ1, TR and PU were combined into a single latent factor, and this three-factor model was compared with the four-factor model.

Structural analyses compared five models using the same data, measurement structure, scaling rules, and estimation settings. Model A specified the sequence AISE → TR → PU → BI while retaining a direct AISE → BI path. Model B reversed the directional relationship between TR and PU. Model C specified parallel TR and PU paths with the TR–PU disturbance covariance fixed at zero. Model C2 retained the parallel paths of Model C while freely estimating the disturbance covariance between TR and PU. Because this specification is just-identified at the structural level, Model C2 reproduces the global fit of the four-factor measurement model; its global fit is therefore not treated as independent evidence of structural robustness. The Extended Model added AISE → PU and TR → BI to Model A. Model evaluation considered absolute fit together with the AIC, BIC, and expected cross-validation index (ECVI) to balance fit, complexity, and relative parsimony. Model C2 was used to estimate the independent TR → BI and PU → BI paths when TR and PU were entered simultaneously. The Extended Model tested whether adding the two auxiliary paths to Model A improved fit; its identical global fit to Model C2 in the present covariance structure is likewise not treated as a separate robustness confirmation.

The serial indirect associations in Models A and B and the specific indirect associations in Models C, C2, and the Extended Model were evaluated with 5000 bootstrap samples, reporting both bias-corrected and percentile 95% confidence intervals (MacKinnon et al., 2004; Preacher & Hayes, 2008). The difference between the unstandardized TR → BI and PU → BI path coefficients was tested within the same Model C2 using consistent latent-variable scaling and the AMOS Model Constraint, with a 5000-sample bootstrap confidence interval. The Bollen–Stine bootstrap was used to assess overall model fit (Bollen & Stine, 1992). χ^2^ difference tests were used only for Model A versus the Extended Model and Model C versus Model C2. Because Model A and Model C2 are not standard nested models, no ordinary χ^2^ difference test was applied between them.

To examine whether the main pattern depended on respondents who selected “Never” on the GenAI-assisted assessment use-frequency item (Q14), a sensitivity analysis excluded those 48 respondents, leaving N = 304. The remaining respondents reported “Rarely,” “Sometimes,” “Often,” or “Always” on Q14. This Q14-based classification reflects self-reported assessment-use frequency and does not indicate the depth, duration, recency, quality, or proficiency of prior experience. The four-factor and TR–PU combined measurement models, Model A, Model C2, bootstrap indirect associations, and the formal TR → BI versus PU → BI coefficient-difference test were re-estimated in this subset. Full sensitivity results are reported in Supplementary Materials, Section S6 (Tables S29–S39).

As an additional check, the five-category Q14 assessment-use-frequency variable was coded from “Never” to “Always” as a single ordered score and entered as an observed covariate in Model C2; full results are reported in Supplementary Materials, Section S7.1 (Tables S40–S43).

Complete model-fit statistics, structural paths, difference tests, and disturbance-covariance results are reported in Supplementary Materials, Section S5 (Tables S20–S28). Additional standardized diagrams for Models B, C, and the Extended Model are provided in Supplementary Materials, Section S8 (Figures S2–S4).

## 4. Results

### 4.1. Sample Characteristics and Preliminary Evidence

The final sample comprised 352 design educators from universities and higher vocational colleges in China. Women represented 52.56% of the sample; 58.52% were aged 26–35 years; 30.68% had 4–6 years of teaching experience; 57.39% held a master’s degree; and 41.76% were lecturers. On the dedicated GenAI-assisted assessment use-frequency item (Q14), 13.64% selected “Never,” whereas 53.98% selected at least “Sometimes.” Thus, based on Q14, the sample included respondents with no prior assessment-related use as well as those reporting some prior assessment-related use, alongside the broader eligibility requirement that all participants could evaluate the assessment context. Full demographic information is provided in Supplementary Materials, Section S2 (Table S7).

Across the 13 core items, means ranged from 2.926 to 4.117 and standard deviations from 0.813 to 0.888. Absolute skewness and kurtosis values were below 0.70, indicating no marked univariate non-normality. Cronbach’s α ranged from 0.809 to 0.874 across the four constructs, and CR ranged from 0.810 to 0.875. Construct-level descriptive statistics, reliability, convergent validity, and correlations are shown in Table 1.

Preliminary factor-structure diagnostics, including the unrotated first-component check, are summarized in Supplementary Materials, Section S3 (Tables S8–S12 and Figure S1). Because this descriptive check cannot rule out common-method variance, the stronger constrained ULMF sensitivity analysis is reported separately in Section 4.7. Conclusions regarding the construct structure are based on confirmatory factor analysis and comparisons of alternative measurement models.

### 4.2. Measurement Evidence for Treating Trust and Perceived Usefulness as Separate Latent Judgments

The four-factor confirmatory measurement model showed excellent fit to the data: χ^2^(59) = 67.075, χ^2^/*df* = 1.137, CFI = 0.996, TLI = 0.994, RMSEA = 0.020, and SRMR = 0.027. Standardized item loadings ranged from 0.730 to 0.831, AVE from 0.587 to 0.637, and CR from 0.810 to 0.875. For every construct, the square root of AVE exceeded its correlations with the other constructs, and the HTMT values ranged from 0.214 to 0.455. Together, these results provided evidence of adequate convergent and discriminant validity. Detailed standardized loadings, AVE, CR, and HTMT results are reported in Supplementary Materials, Section S4 (Tables S13–S19).

To test directly whether TR and PU could be treated as a single construct, a three-factor model was estimated in which the two constructs formed a single latent factor. Fit deteriorated substantially: χ^2^(62) = 353.188, χ^2^/*df* = 5.697, CFI = 0.849, TLI = 0.810, RMSEA = 0.116, and SRMR = 0.091. Standardized loadings for the PU items in the combined factor fell to 0.431–0.513, and the AVE for the combined factor was 0.394. The measurement evidence therefore supports treating TR and PU as separate latent judgments rather than as indicators of a single positive attitude. This comparison addresses RQ1 and provides the measurement basis for RQ2. The two measurement models are compared in Table 2.

### 4.3. Distinct Patterns of Association with Behavioral Intention

All four prespecified paths in Model A were significant: AISE → TR, β = 0.378 (*p* < 0.001); AISE → BI, β = 0.151 (*p* = 0.012); TR → PU, β = 0.443 (*p* < 0.001); and PU → BI, β = 0.414 (*p* < 0.001). H1–H4 were therefore supported in Model A. Model A explained 14.3% of the variance in TR, 19.6% in PU, and 21.5% in BI. These findings indicate that the focal structure is compatible with the data, but they do not imply that educators necessarily form judgments in the sequence AISE → TR → PU → BI. Key path estimates for Model A, Model C2, and the Extended Model are shown in Table 3.

The key evidence for RQ2 comes from Model C2. When TR and PU were entered simultaneously, PU → BI remained positively associated with BI (*B* = 0.366, β = 0.427, *p* < 0.001), whereas TR → BI was close to zero and not significant (*B* = −0.030, β = −0.030, *p* = 0.680). The corresponding path estimates were identical in the Extended Model, but because the two models showed the same global fit in the present covariance structure, they are not treated as independent replications. Using the model constraint with 5000 bootstrap samples, the unstandardized path difference (TR → BI – PU → BI) was −0.396 (*SE* = 0.137, bias-corrected 95% CI [−0.681, −0.142], *p* = 0.001), showing that the unstandardized PU → BI coefficient was significantly larger than the unstandardized TR → BI coefficient. Because TR → BI itself was not significant, its negative point estimate does not support an interpretation of a negative association between TR and BI. Taken together, these findings suggest that PU showed a stronger unique association with BI than TR in the present sample. The full difference test is reported in Supplementary Materials, Table S24. The corresponding bias-corrected 95% bootstrap CIs were [0.237, 0.508] for PU → BI and [−0.207, 0.129] for TR → BI.

### 4.4. Specification Sensitivity of the TR–PU–BI Relations

The candidate structural models were used to examine whether the observed path pattern depended on a particular directional specification. Model A showed good fit and yielded the lowest AIC, BIC, and ECVI among the reported candidate structures, whereas Models B and C fit less well. Model C2 is structurally just-identified and therefore reproduces the global fit of the four-factor CFA. Its global fit is not interpreted as independent support for the substantive conclusion. The Extended Model has the same global fit and information criteria as Model C2 in the present covariance structure and is likewise not treated as a separate fit-based confirmation.

Model C2 remains useful for RQ2 because it estimates TR → BI and PU → BI simultaneously without imposing a directional path between TR and PU. Relative to Model C, allowing the disturbances of TR and PU to covary improved fit (Δχ^2^(1) = 34.840, *p* < 0.001). Adding AISE → PU and TR → BI to Model A did not significantly improve fit in the Extended Model (Δχ^2^(2) = 2.293, *p* = 0.318). Because Models A and C2 are not standard nested models, no ordinary χ^2^ difference test was conducted between them. Fit and information-criterion comparisons are shown in Table 4.

**Figure 2:**
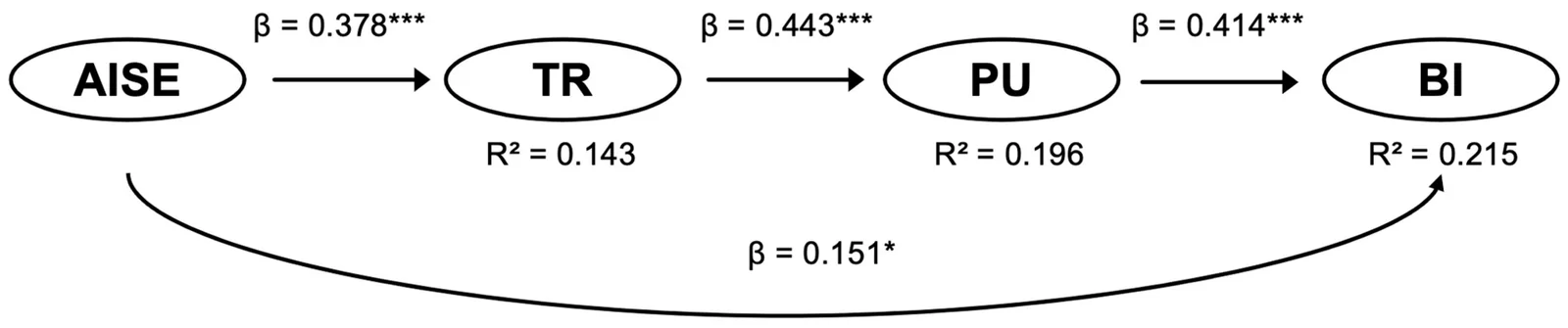
Standardized Results for Model A. Note. Coefficients are standardized estimates; *R*^2^ values indicate explained variance. * *p* < 0.05; *** *p* < 0.001. Model A specifies AISE → TR → PU → BI and retains the direct AISE → BI path.

**Figure 3:**
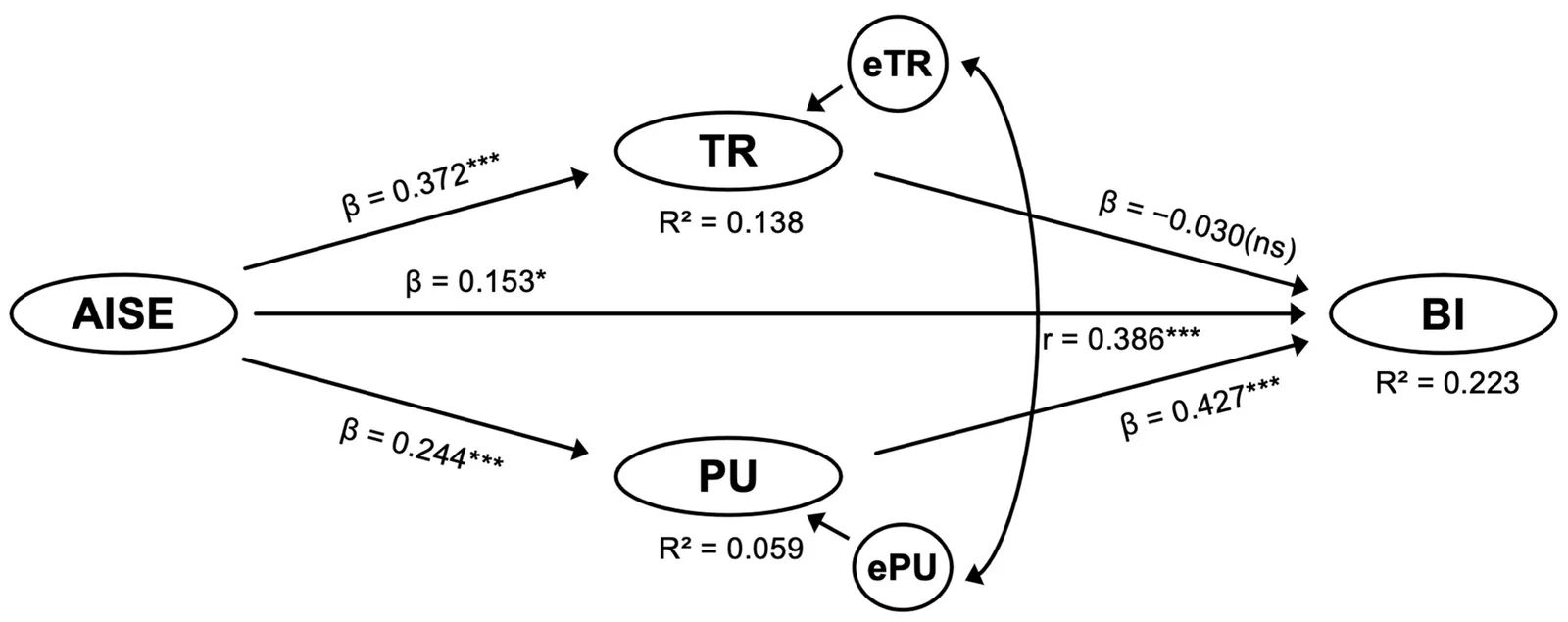
Standardized Results for Model C2. Note. Coefficients are standardized estimates; *R*^2^ values indicate explained variance. The curved double-headed arrow represents the standardized disturbance correlation between eTR and ePU. * *p* < 0.05; *** *p* < 0.001; ns = non-significant. Model C2 simultaneously estimates AISE → TR, AISE → PU, TR → BI, PU → BI, and AISE → BI. eTR and ePU denote the disturbance terms of TR and PU, respectively.

Taken together, the candidate structures cannot establish the temporal order of TR and PU. Model A provides a relatively parsimonious working representation of the observed TR → PU → BI relation, whereas Model C2 allows the two direct associations with BI to be compared without imposing an ordering between TR and PU. The disturbance correlation between TR and PU was significant (r = 0.386, *p* < 0.001). The full unstandardized covariance, bootstrap confidence interval, and nested-model comparisons are reported in Supplementary Materials, Tables S26 and S27. Standardized result diagrams for Model A and Model C2 appear in Figure 2 and Figure 3.

### 4.5. Model-Specific Indirect Associations

Beyond the structural comparisons, the 5000-sample bootstrap results showed a specific serial indirect association of 0.059 for AISE → TR → PU → BI in Model A, with a bias-corrected 95% CI [0.033, 0.098] and a percentile 95% CI [0.031, 0.094]. Neither interval included zero, supporting H5. This result indicates that the specified product of paths is statistically supported in Model A but does not establish a temporally unfolding mediation process. The complete bootstrap results and a summary of hypothesis tests are provided in Tables S23 and S28, respectively.

In Model C2, which does not impose an ordering between TR and PU, the specific indirect association AISE → PU → BI was 0.088, with a bias-corrected 95% CI [0.041, 0.154], whereas AISE → TR → BI was −0.010, with a bias-corrected 95% CI [−0.067, 0.043]. Model Constraint was used to define the difference between AISE → TR → BI – AISE → PU → BI; the difference was −0.098, with a bias-corrected 95% CI [−0.200, −0.019] and a percentile 95% CI [−0.198, −0.018]. Neither interval included zero. The specific indirect association through PU was therefore significantly larger than the corresponding association through TR. These results indicate that, in the present cross-sectional data, the indirect association linking AISE with BI through PU was stronger than the corresponding association through TR. Results for Models A and C2 are summarized in Table 5.

### 4.6. Assessment-Use-Frequency Sensitivity Analysis

After excluding the 48 respondents who selected “Never” on Q14, the sensitivity subset comprised N = 304 respondents. The four-factor measurement model continued to fit well (χ^2^(59) = 66.262, χ^2^/*df* = 1.123, CFI = 0.996, TLI = 0.994, RMSEA = 0.020, SRMR = 0.030), whereas the TR–PU combined model fit substantially worse (χ^2^(62) = 289.578, χ^2^/*df* = 4.671, CFI = 0.860, TLI = 0.824, RMSEA = 0.110, SRMR = 0.086). In Model C2, PU → BI remained significant (B = 0.382, β = 0.432, *p* < 0.001), whereas TR → BI remained nonsignificant (B = −0.050, β = −0.052, *p* = 0.521). The formal unstandardized path difference (TR → BI − PU → BI) was −0.432 (SE = 0.159, bias-corrected 95% CI [−0.777, −0.148], *p* = 0.003), showing that the unstandardized PU → BI coefficient remained significantly larger than the unstandardized TR → BI coefficient in this subset. In Model A, the AISE → TR → PU → BI specific serial indirect association also remained significant (B = 0.059, bias-corrected 95% CI [0.031, 0.101]). Full sensitivity results are provided in Supplementary Materials, Section S6 (Tables S29–S39).

An additional use-frequency covariate analysis yielded the same substantive TR–BI/PU–BI path pattern; full results are reported in Supplementary Materials, Section S7.1 (Tables S40–S43).

### 4.7. Common-Method Sensitivity Analysis

The constrained ULMF model converged normally and showed no negative error variances, standardized estimates greater than 1, or other improper estimates. Its fit was comparable to the original four-factor measurement model: χ^2^(58) = 67.064, χ^2^/*df* = 1.156, CFI = 0.995, TLI = 0.994, RMSEA = 0.021, and SRMR = 0.027. Relative to the original four-factor measurement model, substantive standardized loadings decreased only by approximately 0.011–0.017, AVE by 0.021–0.025, and CR by 0.011–0.016; all remained within acceptable ranges. This sensitivity check did not indicate a material alteration of the substantive four-factor measurement structure; common-source measurement bias nevertheless remains possible. Full fit and measurement-quality comparisons are reported in Supplementary Materials, Section S7.2 (Tables S44 and S45).

## 5. Discussion

### 5.1. From “Whether Educators Accept AI” to Two Assessment Judgments

The measurement evidence supports treating system reliability and practical value as separate judgments, but the central substantive finding is that these judgments are not equally related to BI. In Model C2, PU retained a significant positive association with BI, whereas TR did not show an independent direct association, and the formal path-difference test indicated that the PU → BI association was stronger. The N = 304 use-frequency sensitivity analysis preserved both this path pattern and the formal coefficient difference. The results nevertheless concern acceptance judgments and behavioral intention; they do not demonstrate actual use or assessment effectiveness.

Accordingly, the discussion focuses on the unequal associations of TR and PU with BI rather than on treating either construct as generally more important.

### 5.2. From a General Positive Attitude to Distinct Judgment Functions

This unequal pattern adds nuance to broader technology-acceptance models in which trust and usefulness appear in the same predictive structure without directly comparing their independent associations with BI.

The stronger PU–BI relation is consistent with TAM and recent teacher-focused GenAI research in which perceived work value or performance expectancy is closely related to intention to use (Davis, 1989; Hazzan-Bishara et al., 2025; Khlaif et al., 2024; Y. Liu et al., 2025; Hao et al., 2026). One possible explanation, not directly tested here, is that respondents may have taken anticipated checking or correction effort into account when forming usefulness judgments, even though verification burden was not measured and is not part of the PU item content. The present data cannot establish this mechanism. In addition, the PU and BI items were more directly anchored to respondents’ own assessment work than the TR items, so item-specificity differences remain a plausible alternative explanation for the stronger PU–BI association.

Prior models have placed TR before PU or treated TR as a direct antecedent of BI (Choung et al., 2023; Kılıç & Çelik, 2025; Wu et al., 2011). In the present data, however, TR did not retain an equivalent independent association with BI once PU was modeled simultaneously.

Alternative structural specifications further constrain the interpretation. Model A provides a parsimonious representation of the observed TR → PU → BI relation but does not establish that educators form TR before PU. Model C2 compares TR → BI and PU → BI without imposing this ordering; because it is structurally just-identified, its global fit is not treated as independent evidence.

AISE provides a different layer of context. Its positive associations with TR and BI indicate that educators’ beliefs about their own ability to operate GenAI and engage with assessment-related tasks are associated with both trust and intention to use. The smaller but significant direct AISE–BI relation suggests that this capability belief is not fully represented by TR and PU. However, AISE remains a self-judgment rather than an objective measure of AI literacy, error-detection ability, or professional verification performance.

Overall, the contribution is a focused comparison of the unequal associations of TR and PU with BI in this assessment-related context. The results do not establish a design-specific mechanism, a fixed TR → PU sequence, or a causal process.

### 5.3. From “Building Trust” to Task-Specific Value and Teacher Review

Because the present study did not test a trust-building intervention or a particular assessment workflow, the practical implications are best treated as directions for future implementation research. Future institutional trials could distinguish reliability judgments from task-specific value judgments while preserving educator review and final decision-making authority.

One practical direction is to evaluate GenAI on concrete assessment tasks rather than on general feature demonstrations. Institutions might examine tasks such as organizing criteria, preparing draft feedback, summarizing student performance, or supporting self-assessment, while recording the checking and revision required. Such trials could make perceived task value more observable, but the present study did not test whether GenAI actually reduces workload or improves feedback quality.

A second direction is calibrated reliance rather than simply increasing trust. Implementation studies could examine whether educators can be supported in identifying when outputs may be consulted, where uncertainty or common errors arise, and when independent verification is needed. Making output provenance, limitations, and subsequent revisions traceable could then be evaluated as potential support for this process (Lee & See, 2004; McGrath et al., 2025); these interface and institutional arrangements require direct evaluation in future studies. Calibrated reliance itself was not measured as a construct or tested as an outcome in the present survey; it is used here only as a direction for future implementation research.

At the institutional level, future implementation studies could examine arrangements that preserve explicit educator authority to review outputs and make final decisions. In design assessment, assistive uses such as organizing feedback or summarizing process materials may be plausible starting points, whereas judgments involving work intent, aesthetic expression, or contextual fit may warrant greater educator interpretation. These design-specific task boundaries were not measured in the present study and should therefore be treated as hypotheses for future implementation research rather than as findings from the current survey.

## 6. Conclusions

This study examined the relationships among AISE, TR, PU, and BI in the intentions of higher-education design teachers to use GenAI for assessment-related work in China. Measurement evidence supported treating TR and PU as separate latent judgments, and their associations with BI were not equivalent: PU showed the stronger independent association, whereas TR did not retain a significant direct relation when both were modeled simultaneously. The N = 304 use-frequency sensitivity analysis preserved the significant TR → BI versus PU → BI coefficient difference. These results concern acceptance judgments and intention to use, not actual adoption or assessment outcomes.

Taken together, the findings indicate that reliability and perceived task value are related but nonredundant judgments in this assessment-related context.

The study remains limited by its cross-sectional design, self-report measures, and purposive nonprobability sample. The findings cannot establish the temporal order or causal direction between TR and PU, nor can BI be equated with actual use, sustained use, or assessment quality. Although the N = 304 sensitivity analysis excluded respondents who selected “Never” on the dedicated assessment-use-frequency item (Q14), prior experience was operationalized using this self-reported frequency item rather than measures of the depth, quality, duration, or recency of use; expectation-based and experience-based judgments therefore cannot be fully disentangled. The TR items were framed relatively generally around reliability and trustworthiness, whereas the PU and BI items were more directly tied to respondents’ own assessment work. This difference in item specificity may have contributed to the stronger PU–BI association and cannot be ruled out by the present data. The constrained ULMF sensitivity analysis produced only small changes in substantive loadings, AVE, and CR and showed no improper estimates, indicating that the four-factor measurement structure was not materially altered when a constrained common-method factor was introduced. However, this post hoc diagnostic cannot eliminate common-method concerns, and common-source self-report data remain vulnerable to influences such as general positive attitudes, social desirability, or response habits (Podsakoff et al., 2003; Richardson et al., 2009). The questionnaire did not directly measure the verification of visual evidence, understanding of creative processes, aesthetic judgment, or design intent, and no comparison group from other disciplines was included; the observed pattern therefore cannot be interpreted as specific to design educators. Recruitment through professional groups, disciplinary networks, academic communities, and peer referrals may also have introduced self-selection; accordingly, the sample should not be treated as representative of Chinese design educators as a population.

Future studies should therefore use longitudinal tracking, cross-lagged designs, or experiments to examine how the two judgments form and change, and combine platform logs, classroom observation, task performance, and output-verification records to assess actual behavior. Cross-disciplinary or cross-cultural work should first establish measurement comparability and then examine when conditions such as task complexity, training experience, institutional support, or algorithmic transparency alter the associations among TR, PU, and BI.

## Figures and Tables

**Table 1:** Construct-Level Descriptive Statistics, Reliability, and Correlations.

Construct	*M*	*SD*	α	CR	AVE	AISE	TR	PU	BI
AISE	3.434	0.728	0.874	0.875	0.637	0.798			
TR	3.205	0.733	0.826	0.826	0.614	0.316 **	0.783		
PU	3.336	0.740	0.809	0.810	0.587	0.206 **	0.358 **	0.766	
BI	3.910	0.699	0.812	0.813	0.592	0.211 **	0.176 **	0.369 **	0.769

Note. Diagonal entries are square roots of AVE; off-diagonal entries are Pearson correlations. AISE = assessment-context AI self-efficacy; TR = trust in GenAI-assisted assessment; PU = perceived usefulness of GenAI-assisted assessment; BI = behavioral intention to use GenAI-assisted assessment. ** *p* < 0.01.

**Table 2:** Comparison of the Four-Factor and TR–PU Combined Measurement Models.

Model	χ^2^ (*df*)	χ^2^/*df*	CFI	TLI	RMSEA	SRMR
Four-factor	67.075 (59)	1.137	0.996	0.994	0.020	0.027
Three-factor (TR + PU)	353.188 (62)	5.697	0.849	0.81	0.116	0.091

Note. The four-factor model specifies AISE, TR, PU, and BI as separate latent variables. The TR + PU model combines TR and PU into one latent factor.

**Table 3:** Key Path Estimates for Model A, Model C2, and the Extended Model.

Model	Path	*B*	β	*SE*	C.R.	*p*
A	AISE → TR	0.329	0.378	0.055	5.938	<0.001
A	AISE → BI	0.128	0.151	0.051	2.509	0.012
A	TR → PU	0.504	0.443	0.076	6.642	<0.001
A	PU → BI	0.354	0.414	0.058	6.094	<0.001
C2	AISE → TR	0.323	0.372	0.056	5.824	<0.001
C2	AISE → PU	0.242	0.244	0.063	3.857	<0.001
C2	AISE → BI	0.130	0.153	0.056	2.340	0.019
C2	PU → BI	0.366	0.427	0.066	5.574	<0.001
C2	TR → BI	−0.030	−0.030	0.072	−0.413	0.68
Extended	AISE → PU	0.093	0.094	0.064	1.440	0.15
Extended	TR → BI	−0.030	−0.030	0.072	−0.413	0.68

Note. *B* = unstandardized coefficient; β = standardized coefficient. Model C2 estimates the independent TR → BI and PU → BI relations when TR and PU enter simultaneously. For the Extended Model, only the two added auxiliary paths are shown. Complete path estimates and *R*^2^ values for all five candidate models are provided in Supplementary Materials, Table S22. For the three principal direct paths in Model C2, bias-corrected 95% bootstrap CIs for unstandardized B were [0.237, 0.508] for PU → BI, [−0.207, 0.129] for TR → BI, and [0.012, 0.254] for AISE → BI.

**Table 4:** Comparison of the Focal, Alternative, and Extended Structural Models.

Model	χ^2^ (*df*)	CFI	RMSEA	SRMR	AIC	BIC	ECVI
A: TR → PU	69.368 (61)	0.996	0.020	0.033	129.368	245.277	0.369
B: PU → TR	123.426 (61)	0.968	0.054	0.085	183.426	299.334	0.523
C: strict independent parallel	101.915 (60)	0.978	0.045	0.072	163.915	283.687	0.467
C2: correlated parallel	67.075 (59)	0.996	0.020	0.027	131.075	254.711	0.373
Extended	67.075 (59)	0.996	0.020	0.027	131.075	254.711	0.373

Note. All models retain the direct AISE → BI path. Model C fixes the disturbance covariance between TR and PU at zero; Model C2 freely estimates this covariance. Model C2 and the Extended Model show identical global fit and identical information criteria in the present data and are therefore not treated as independent confirmations. Full fit indices, including TLI, RMSEA 90% CI, and Bollen–Stine *p*, are reported in Supplementary Materials, Table S20. Relative to Model A, ΔAIC/ΔBIC were 54.058/54.057 for Model B, 34.547/38.410 for Model C, and 1.707/9.434 for both Model C2 and the Extended Model.

**Table 5:** Model-Specific Indirect Associations in Models A and C2.

Model	Specific Indirect Path	Estimate	Bias-Corrected 95% CI	Percentile 95% CI	Result
A	AISE → TR → PU → BI	0.059	[0.033, 0.098]	[0.031, 0.094]	Significant
C2	AISE → TR → BI	−0.010	[−0.067, 0.043]	[−0.067, 0.043]	Not significant
C2	AISE → PU → BI	0.088	[0.041, 0.154]	[0.039, 0.151]	Significant
C2	TR indirect − PU indirect	−0.098	[−0.200, −0.019]	[−0.198, −0.018]	Significant difference

Note. Estimates are unstandardized indirect effects; in the main text, they are interpreted as cross-sectional indirect associations. The difference is defined as AISE → TR → BI minus AISE → PU → BI. The indirect-association difference test is reported in Supplementary Materials, Table S25.

## Data Availability

The data supporting the findings of this study are available from the corresponding author upon reasonable request. The data are not publicly available because of participant privacy considerations.

## Supplementary Materials

The following supporting information can be downloaded at https://www.mdpi.com/article/10.3390/bs16101930/s1. The accompanying Supplementary Materials file contains full-scale definitions and items, the Chinese-language adaptation and back-translation workflow, data screening and sample characteristics, item-level and measurement-model results, complete structural and bootstrap analyses, the assessment-use-frequency sensitivity analysis, the use-frequency covariate sensitivity analysis, the constrained ULMF common-method sensitivity analysis, and supplementary figures (Tables S1–S45 and S5A–C; Figures S1–S4).

## Abbreviations

The following abbreviations are used in this manuscript:

AI	Artificial intelligence
GenAI	Generative artificial intelligence
AISE	Assessment-context AI self-efficacy
TR	Trust in GenAI-assisted assessment
PU	Perceived usefulness
BI	Behavioral intention
TAM	Technology acceptance model
UTAUT	Unified Theory of Acceptance and Use of Technology
TPB	Theory of Planned Behavior
GAICS-TR	Generative Artificial Intelligence Competency Self-Efficacy Scale for Teachers and Researchers
S-TIAS	Short Form of the Trust in Automation Scale
HTMT	Heterotrait–monotrait ratio
RMSEA	Root mean square error of approximation
SRMR	Standardized root mean square residual
KMO	Kaiser–Meyer–Olkin measure of sampling adequacy
CFI	Comparative fit index
TLI	Tucker–Lewis index
CR	Composite reliability
AVE	Average variance extracted
AIC	Akaike information criterion
BIC	Bayesian information criterion
ECVI	Expected cross-validation index
CI	Confidence interval
SE	Standard error
C.R.	Critical ratio
IRB	Institutional review board
PII	Personally identifiable information
RQ	Research question
ULMF	Unmeasured latent method factor
