# Supplementary material for: Trust and Perceived Usefulness in GenAI-Assisted Assessment Adoption Among Design Teachers in Higher Education: Distinct Judgments and Their Associations with Behavioral Intention
Source: Behav Sci (Basel). 2026 Oct 17;16(10):1930. doi: 10.3390/bs16101930 (PMC13701679; doi:10.3390/bs16101930)
Supplement: Supplementary file 1 [file behavsci-16-01930-s001.zip › behavsci-4584437-supplementary.pdf]

Supplementary Materials

These Supplementary Materials accompany the main manuscript. Unless otherwise stated, statistical results are based on the final valid sample (N = 352). Section S6 reports a Q14-defined prior-use sensitivity subset (N = 304) formed by excluding the 48 respondents who selected "Never" on the dedicated GenAI-assisted assessment use-frequency item (Q14). Section S7 reports the additional use-frequency covariate sensitivity analysis and the constrained ULMF common-method sensitivity analysis. Analyses were conducted in SPSS 28.0 and AMOS 27.0, with 5,000 bootstrap resamples where reported.

The questionnaire was administered in Chinese. The original survey sequence is reported as Q1–Q18: Q1 = informed consent; Q2–Q3 = screening items; Q4–Q16 = background items; Q17 = the 13-item core measurement matrix; and Q18 = an optional open-response item. Item IDs AISE1–AISE4, TR1–TR3, PU1–PU3, and BI1–BI3 identify the individual scale items. Tables S2–S5 present the final Chinese items administered and the reconciled verified English versions established after two independent blinded back-translations.

Full bibliographic details for sources cited in these Supplementary Materials are provided in the References section at the end of this file and, where applicable, in the main manuscript.

S1. Measures, construct definitions, and items

Tables S1–S5 provide the construct definitions, source measures, and all 13 final core items from Q17, with the administered Chinese wording and the reconciled verified English versions established after two independent blinded back-translations.

Table S1. Definitions, item counts, and primary sources of the core constructs

Construct	Operational definition	No. of items	Primary source and contextual adaptation
Assessment-context AI self-efficacy (AISE)	Educators' judgment of whether they can use GenAI to complete assessment-related tasks, support student self-assessment, improve assessment approaches, and analyze student performance.	4	Primarily informed by assessment-related content in the GAICS-TR (Xia et al., 2026) and adapted to higher education design assessment.
Trust (TR)	The extent to which educators judge GenAI-assisted assessment tools and their outputs to be trustworthy, stable, and suitable as a reference for assessment judgment.	3	Adapted from the S-TIAS (McGrath et al., 2025), with contextualization informed by automation-trust concepts in Jian et al. (2000).
Perceived usefulness (PU)	The extent to which educators judge GenAI-assisted assessment to improve efficiency, enhance assessment quality, and help complete assessment tasks.	3	Adapted from the perceived usefulness scale of Davis (1989).
Behavioral intention (BI)	Educators' willingness to use or continue using GenAI-assisted assessment and to prioritize it when conditions permit.	3	Informed by the behavioral-intention measure of Venkatesh et al. (2003).

Note. All core items used a five-point Likert scale (1 = strongly disagree, 2 = disagree, 3 = neutral, 4 = agree, 5 = strongly agree). All items were positively keyed; no reverse scoring was required.

Table S2. Assessment-context AI self-efficacy (AISE) items

Item (Q17)	Questionnaire wording
AISE1	I can use generative AI to assist me in completing teaching-assessment tasks.<br>我能够使用生成式 AI辅助完成教学评价相关任务。
AISE2	I can use generative AI to support students in self-assessment or reflective assessment activities.<br>我能够使用生成式 AI支持学生开展自我评价或反思性评价活动。
AISE3	I can use generative AI to design or improve teaching-assessment approaches and processes.<br>我能够借助生成式 AI设计或优化教学评价方式与评价流程。
AISE4	I can use generative AI to analyze student learning performance and generate useful input for assessment.<br>我能够借助生成式 AI分析学生学习表现，并形成有价值的评价参考。

Table S3. Trust (TR) items

Item (Q17)	Questionnaire wording
TR1	Overall, I trust generative AI tools used to support assessment.<br>我总体上信任生成式 AI辅助评价工具。
TR2	I believe generative AI can operate stably and consistently when supporting assessment.<br>我认为生成式 AI在评价支持中能够稳定且一致地运行。
TR3	I believe the outputs generated by generative AI can be used as a reference for assessment judgments.<br>我认为生成式 AI生成的结果可以作为评价判断的参考。

Table S4. Perceived usefulness (PU) items

Item (Q17)	Questionnaire wording
PU1	Using generative AI to support assessment can improve my assessment-work efficiency.<br>使用生成式 AI辅助评价能够提高我的教学评价工作效率。
PU2	Using generative AI to support assessment can improve the quality of my teaching assessment.<br>使用生成式 AI辅助评价能够提升我的教学评价质量。
PU3	Overall, generative AI-assisted assessment is helpful for my teaching-assessment work.<br>总体而言，生成式 AI辅助评价对我的教学评价工作是有帮助的。

Table S5. Behavioral intention (BI) items

Item (Q17)	Questionnaire wording
BI1	I am willing to use generative AI-assisted assessment in the future.<br>我愿意在未来使用生成式 AI辅助评价。
BI2	I plan to continue using generative AI-assisted assessment in my future teaching assessment.<br>我计划在今后的教学评价中持续使用生成式 AI辅助评价。
BI3	When conditions permit, I will give priority to using generative AI-assisted assessment.<br>在条件允许的情况下，我会优先考虑使用生成式 AI辅助评价。

S1.1. Chinese-language adaptation, pilot testing, and back-translation

The research team first reviewed the English-language source or source-informed items and established context-adapted English reference wording for GenAI-assisted assessment among higher education design teachers while retaining the intended content of each construct. The first author then translated the context-adapted items into Chinese. ChatGPT was used only for bilingual wording checks and linguistic refinement during the initial translation stage; it did not determine construct content, scoring, or statistical results. All wording decisions were reviewed and

confirmed by the researchers before the Chinese pilot. Contextual adaptation extended beyond literal translation: AISE1–AISE4 describe perceived capability for specific assessment tasks; TR1–TR3 frame overall trust, stability/consistency, and outputs as assessment references; PU1–PU3 frame efficiency, assessment quality, and task helpfulness; and BI1–BI3 frame future use, continued use, and priority when conditions permit.

Before the formal survey, 12 higher education design educators completed a Chinese pilot questionnaire. The pilot was used to assess scenario clarity, item wording, questionnaire flow, possible repetition, and approximate completion time. Structured feedback questions addressed the clarity of the assessment scenario, difficult sections, potentially repetitive items, and approximate completion time. All 12 selected the current-user option in the pilot screening question. Their recorded responses to these structured feedback questions are summarized in Table S5A. Pilot feedback questions P1–P4 are separate from Q1–Q18 in the main survey.

Table S5A. Documented feedback from the 12-person Chinese pilot

Feedback question	Recorded responses
Scenario clarity (P1)	Very clear: 3; basically clear: 7; moderate: 2; unclear or very unclear: 0.
Most difficult section (P2)	All sections relatively easy: 11; scenario explanation: 1; individual AISE, TR, PU, or BI sections: 0.
Possible repetition (P3; optional)	One participant explicitly reported no obvious repetition; the other 11 left this item blank.
Approximate completion time (P4)	Under 3 min: 1; 3–5 min: 9; 5–8 min: 2; over 8 min: 0.

Note. Figures describe the 12 retained pilot forms only. Blank responses to the optional question were not interpreted as evidence that no problems occurred. In addition, all 12 pilot respondents selected the current-user option, and BI2 used continued-use wording; the pilot therefore did not directly assess how respondents with no prior assessment-related GenAI use understood that item.

Following the pilot and subsequent wording refinements, two independent Chinese–English bilingual experts with design backgrounds, neither of whom had participated in the initial translation or contextual adaptation, independently back-translated the candidate Chinese items. Neither expert was given access to the original source items or the predetermined context-adapted English reference wording during back-translation. The research team compared both back-translations with the context-adapted English reference wording and, where necessary, the source-scale wording, focusing on construct meaning, assessment context, and semantic scope. Discrepancies were reviewed and reconciled before the final Chinese version was confirmed. All four AISE items were revised between the pilot and final versions; the nine TR, PU, and BI Chinese items remained unchanged (Table S5B). Tables S2–S5 present the final administered Chinese items together with the reconciled verified English versions established after the two blinded back-translations. These English versions correspond to the final administered Chinese items and should not be interpreted as verbatim reproductions of the original source-scale items.

Table S5B. Wording changes between the pilot and final Chinese questionnaires

Item	Pilot Chinese wording (with English translation)	Final Chinese wording (with English translation)
AISE1	我能够使用生成式 AI完成教学评价相关任务。<br>I can use GenAI to complete assessment-related tasks in teaching.	我能够使用生成式 AI辅助完成教学评价相关任务。<br>I can use generative AI to assist me in completing teaching-assessment tasks.
AISE2	我能够使用生成式 AI支持学生开展自我评价活动。<br>I can use GenAI to support students in self-assessment activities.	我能够使用生成式 AI支持学生开展自我评价或反思性评价活动。<br>I can use generative AI to support students in self-assessment or reflective assessment activities.
AISE3	我能够借助生成式 AI设计或优化评价方式与评价流程。<br>I can use GenAI to design or optimize assessment methods and processes.	我能够借助生成式 AI设计或优化教学评价方式与评价流程。<br>I can use generative AI to design or improve teaching-assessment approaches and processes.
AISE4	我能够借助生成式 AI对学生学习表现进行评价与判断。<br>I can use GenAI to evaluate and judge students' learning performance.	我能够借助生成式 AI分析学生学习表现，并形成有价值的评价参考。<br>I can use generative AI to analyze student learning performance and generate useful input for assessment.

Note. Table S5B records the wording changes between the pilot and final Chinese questionnaires; individual revisions cannot be attributed to specific pilot comments.

The pilot assessed comprehensibility and questionnaire administration, and the two independent blinded back-translations served as semantic-consistency checks. Neither procedure constitutes evidence of cross-language measurement invariance, and no reliability or validity coefficient was calculated from the 12-person pilot. Final-sample psychometric results are reported in Sections S3–S4. Table S5C summarizes the three-stage adaptation and verification workflow.

Table S5C. Summary of the Chinese-language adaptation and back-translation workflow

Stage	Procedure	Interpretive boundary
1. Initial translation and wording check	The research team reviewed the source or source-informed English items and established context-adapted English reference wording. The first author translated the context-adapted items into Chinese. ChatGPT was used only for bilingual wording checks and linguistic refinement, followed by researcher review before the pilot.	Produced the candidate Chinese pilot wording; this stage did not constitute psychometric validation.
2. Chinese pilot	Twelve higher education design educators from multiple design disciplines completed the Chinese pilot, which was used to assess scenario clarity, item wording, difficult sections, possible repetition, questionnaire flow, and approximate completion time.	Used to assess comprehensibility and administration only; no reliability, validity, or measurement-invariance inference was drawn from the pilot.
3. Independent back-translation and reconciliation	Two independent Chinese–English bilingual experts with design backgrounds, blind to the original source items and the predetermined context-adapted English reference wording and uninvolved in the initial translation or contextual adaptation, independently back-translated the candidate Chinese items. The research team compared both back-translations with the context-adapted English reference wording and, where necessary, the source-scale wording, and reconciled discrepancies before finalization.	Confirmed semantic consistency for the final Chinese version. AISE1–AISE4 were revised; the Chinese wording of TR1–TR3, PU1–PU3, and BI1–BI3 remained unchanged.

Note. This workflow documents translation/adaptation and semantic verification only. It should not be interpreted as evidence of cross-language measurement equivalence. The item-level final Chinese wording and the reconciled verified English versions established after two independent blinded back-translations are reported in Tables S2–S5; Table S5B records the Chinese wording changes between the pilot and final versions.

S2. Data screening and sample characteristics

Tables S6 and S7 document data screening and the composition of the final sample.

Question 1 (Q1) addressed informed consent. Only respondents who provided consent could proceed to the questionnaire. Q1 was not included in the statistical analysis.

Table S6. Data-cleaning flow

Step	Exclusion criterion	Excluded	Remaining
Remove clearly invalid responses	All 13 core measurement items missing (n = 4); records flagged as duplicates (n = 4); identical ratings on all 13 core items (n = 4).	12	388
Remove ineligible responses	No relevant teaching-assessment experience (Q2; n = 10) and/or insufficient understanding of the GenAI-assisted assessment scenario (Q3, option C [export value 3]; n = 10); five met both criteria.	15	373
Remove low-quality responses	Completion time < 60 seconds (n = 3); partial core-item completion (6/13 and 9/13 items completed; n = 2); repeated ascending/descending response sequences across the 13 core items (n = 2).	7	366
Remove logically inconsistent responses	Conservative three-item logical-consistency rule: exclusion required the simultaneous occurrence of (a) Q3 = 2, indicating that the respondent had not actually used GenAI-assisted assessment but could judge the scenario; (b) Q14 = 5 ("Always" for GenAI-assisted assessment use); and (c) Q16 = 5 ("Very familiar, with relatively frequent use"). Isolated discrepancies between these items did not trigger exclusion. Q13 was used only as an auxiliary consistency check and did not independently determine exclusion.	14	352
Final valid sample	Used for formal statistical analysis	—	352

Note. Screening was sequential, and each response was counted once at the first applicable stage. The three responses below the 60-second threshold took 24, 31, and 43 seconds. All 14 logically inconsistent responses met the complete three-item pattern: Q3 = 2 (the option indicating that the respondent had not actually used GenAI-assisted assessment but could judge the scenario), Q14 = 5 ("Always" for GenAI-assisted assessment use), and Q16 = 5 ("Very familiar, with relatively frequent use"). Isolated discrepancies between these items were retained and did not trigger exclusion. Q13 = 1 ("Never" for GenAI use in everyday teaching) was examined only as an auxiliary consistency check and did not independently determine exclusion. Non-use alone did not trigger exclusion. Overall, 48 of 400 responses were excluded, leaving 352 (88.0%). These screening criteria were applied before the confirmatory and structural analyses reported in the manuscript.

Table S7. Demographic and GenAI-use characteristics of the sample ($N = 352$)

Variable	Category	*n*	%
Gender	Male	153	43.47
Gender	Female	185	52.56
Gender	Prefer not to disclose	14	3.98
Age	Under 25	36	10.23
Age	26–30 years	104	29.55
Age	31–35 years	102	28.98
Age	36–40 years	57	16.19
Age	41–45 years	32	9.09
Age	46 years or older	21	5.97
Teaching experience	Less than 1 year	21	5.97
Teaching experience	1–3 years	70	19.89

Variable	Category	*n*	%
Teaching experience	4–6 years	108	30.68
Teaching experience	7–10 years	70	19.89
Teaching experience	11–15 years	51	14.49
Teaching experience	16 years or more	32	9.09
Region	East China	66	18.75
Region	South China	66	18.75
Region	North China	53	15.06
Region	Central China	53	15.06
Region	Southwest China	30	8.52
Region	Northwest China	20	5.68
Region	Northeast China	39	11.08
Region	Other	25	7.10
Highest degree	Bachelor's	41	11.65
Highest degree	Master's	202	57.39
Highest degree	Doctorate	93	26.42
Highest degree	Other	16	4.55
Academic rank	Teaching assistant	58	16.48
Academic rank	Lecturer	147	41.76
Academic rank	Associate professor	86	24.43
Academic rank	Professor	42	11.93
Academic rank	Other	19	5.40
Institution type	Comprehensive university	85	24.15
Institution type	Arts institution	109	30.97
Institution type	Science/engineering institution	70	19.89
Institution type	Higher vocational college	52	14.77
Institution type	Other	36	10.23
Design discipline	Visual communication/graphic design	86	24.43
Design discipline	Product design	36	10.23
Design discipline	Environmental/architecture/interior design	51	14.49
Design discipline	Animation/digital media	53	15.06
Design discipline	Fashion/apparel design	39	11.08
Design discipline	Interaction/service design	59	16.76
Design discipline	Other	28	7.95
GenAI-related training	None	78	22.16
GenAI-related training	Lecture/short-term training	194	55.11
GenAI-related training	More systematic training	80	22.73
Frequency of GenAI use in everyday teaching	Never	37	10.51
Frequency of GenAI use in everyday teaching	Rarely	86	24.43

Variable	Category	*n*	%
Frequency of GenAI use in everyday teaching	Sometimes	131	37.22
Frequency of GenAI use in everyday teaching	Often	72	20.45
Frequency of GenAI use in everyday teaching	Always	26	7.39
Frequency of GenAI-assisted assessment use	Never	48	13.64
Frequency of GenAI-assisted assessment use	Rarely	114	32.39
Frequency of GenAI-assisted assessment use	Sometimes	121	34.38
Frequency of GenAI-assisted assessment use	Often	50	14.20
Frequency of GenAI-assisted assessment use	Always	19	5.40
Position/employment type	Full-time faculty	215	61.08
Position/employment type	Part-time/adjunct faculty	47	13.35
Position/employment type	Externally appointed instructor	36	10.23
Position/employment type	Postdoctoral researcher/researcher with teaching duties	30	8.52
Position/employment type	Other	24	6.82
Level of GenAI familiarity/exposure	Completely unfamiliar	0	0.00
Level of GenAI familiarity/exposure	Limited awareness, no actual use	96	27.27
Level of GenAI familiarity/exposure	Some understanding, occasional exposure	131	37.22
Level of GenAI familiarity/exposure	Good understanding, with relevant use experience	96	27.27
Level of GenAI familiarity/exposure	Very familiar, with relatively frequent use	29	8.24

Note. Percentages are based on the final valid sample (N = 352). The "Under 25" category reproduces the original questionnaire wording; the target population comprised adult higher-education design educators. Totals may differ slightly from 100% because of rounding. Background variables in Table S7 correspond to Q4–Q16 in survey order; GenAI-assisted assessment use frequency is Q14, and GenAI familiarity/exposure is Q16. Q16 assesses general GenAI familiarity/exposure and was not used to define prior GenAI-assisted assessment use in Section S6; the Section S6 sensitivity subset was operationalized from the dedicated assessment-use-frequency item Q14. Because recruitment was purposive and network-based, these distributions describe the analytical sample and should not be interpreted as population-representative estimates for Chinese design educators.

S3. Item descriptives, reliability, and preliminary factor analysis

Tables S8–S12 report item descriptives, reliability, and preliminary factor-analysis results; Figure S1 shows the preliminary principal-component scree plot.

Table S8. Descriptive statistics for the core items

Item	*N*	*M*	*SD*	Skewness	Kurtosis
AISE1	352	3.4858	.85723	−.024	−.372
AISE2	352	3.5682	.84118	−.057	−.437
AISE3	352	2.9261	.83390	−.157	−.505
AISE4	352	3.7557	.88512	−.295	−.615
TR1	352	3.5653	.84141	−.279	.049

Item	N	M	SD	Skewness	Kurtosis
TR2	352	3.0142	.88824	−.200	−.440
TR3	352	3.0369	.82434	−.038	.050
PU1	352	3.4716	.87660	−.308	−.025
PU2	352	3.0028	.87217	−.213	−.363
PU3	352	3.5341	.85999	−.268	−.216
BI1	352	4.0085	.81471	−.588	.268
BI2	352	4.1165	.81339	−.664	.074
BI3	352	3.6051	.83395	−.218	−.196

Note. Standard errors of skewness and kurtosis were .130 and .259, respectively.

Table S9. Item-level internal-consistency results

Construct	Item	CITC	α if item deleted	Construct Cronbach's α
AISE	AISE1	.765	.824	.874
AISE	AISE2	.743	.834	
AISE	AISE3	.677	.859	
AISE	AISE4	.735	.837	
TR	TR1	.658	.783	.826
TR	TR2	.697	.745	
TR	TR3	.694	.749	
PU	PU1	.677	.718	.809
PU	PU2	.631	.766	
PU	PU3	.665	.731	
BI	BI1	.665	.738	.812
BI	BI2	.686	.717	
BI	BI3	.634	.770	

Note. CITC = corrected item–total correlation; α = Cronbach's alpha. Construct-level α is shown on the first row of each construct.

Table S10. Summary of preliminary factor-analysis diagnostics

Indicator	Result
KMO	.818
Bartlett's test of sphericity χ^2	1974.854
df	78
p	< .001
Components with eigenvalue > 1	4
Cumulative variance explained	73.195%
Variance explained by unrotated first component	33.294%

Note. KMO = Kaiser–Meyer–Olkin measure of sampling adequacy. The unrotated first component explained 33.294% of the total variance (also reported in Table S11). This first-component diagnostic is only a limited post-hoc descriptive check and cannot rule out common-method bias. No a priori marker variable was included in the questionnaire. A stronger constrained ULMF sensitivity analysis is reported in Section S7.2 (Tables S44–S45).

Table S11. Total variance explained in the principal component analysis

Component	Initial eigenvalue	Initial %	Cumulative %	Extraction eigenvalue	Extraction %	Extraction cumulative %	Rotated sum of squares	Rotated %	Rotated cumulative %
1	4.328	33.294	33.294	4.328	33.294	33.294	2.913	22.405	22.405
2	2.202	16.940	50.233	2.202	16.940	50.233	2.237	17.210	39.615
3	1.759	13.532	63.765	1.759	13.532	63.765	2.197	16.896	56.512
4	1.226	9.430	73.195	1.226	9.430	73.195	2.169	16.684	73.195
5	.540	4.150	77.345						
6	.486	3.742	81.087						
7	.419	3.219	84.306						
8	.407	3.128	87.435						
9	.372	2.861	90.295						
10	.357	2.746	93.042						
11	.331	2.547	95.589						
12	.300	2.307	97.896						
13	.273	2.104	100.000						

Table S12. Component loadings after Varimax rotation

Item	Component 1 (AISE)	Component 2 (TR)	Component 3 (BI)	Component 4 (PU)
AISE1	.874			
AISE2	.835			
AISE3	.802			
AISE4	.837			
TR1		.830		
TR2		.819		
TR3		.853		
PU1				.812
PU2				.795
PU3				.849
BI1			.839	
BI2			.850	
BI3			.807	

Note. Extraction used principal component analysis with Varimax orthogonal rotation and Kaiser normalization. Only loadings with absolute values ≥ .50 are shown.

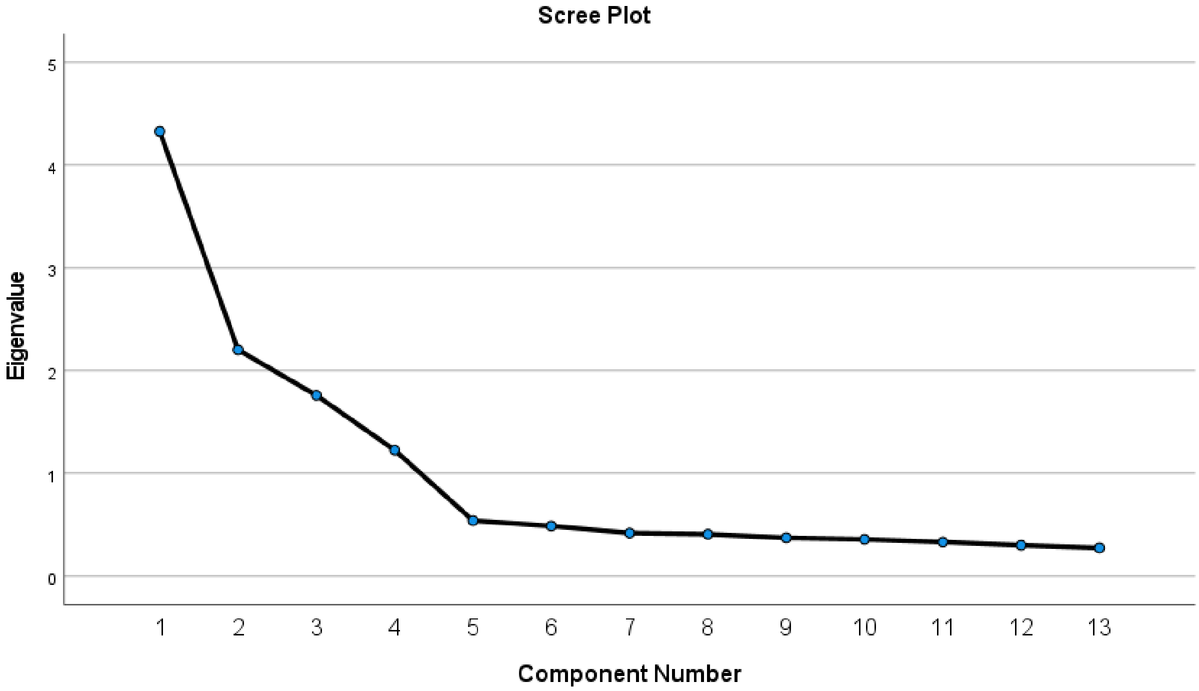

Figure S1. Preliminary principal-component scree plot

Note. The figure is provided only as a preliminary structural diagnostic. Formal construct evaluation is based on CFA and alternative measurement-model comparisons.

S4. Full measurement-model and discriminant-validity results

Tables S13–S19 report measurement-model fit, factor loadings, and discriminant-validity results used to evaluate whether TR and PU should be modeled as separate latent judgments in the subsequent structural analyses.

Table S13. Fit comparison of the four-factor and TR–PU combined measurement models

Model	χ^2	*df*	χ^2/df	NFI	IFI	TLI	CFI	GFI	RMSEA	SRMR
Four-factor M1	67.075	59	1.137	.967	.996	.994	.996	.971	.020	.027
Three-factor M2 (TR+PU)	353.188	62	5.697	.824	.850	.810	.849	.838	.116	.091

Note. M1 specifies AISE, TR, PU, and BI as separate latent variables; M2 combines TR and PU. NFI = normed fit index; IFI = incremental fit index; TLI = Tucker–Lewis index; CFI = comparative fit index; GFI = goodness-of-fit index; RMSEA = root mean square error of approximation; SRMR = standardized root mean square residual.

Table S14. Confirmatory factor analysis results for the four-factor measurement model (M1)

Latent variable	Item	Standardized loading	*SE*	AVE	CR
AISE	AISE1	.831	—	.637	.875
AISE	AISE2	.816	.057		
AISE	AISE3	.733	.058		
AISE	AISE4	.808	.060		
TR	TR1	.739	—	.614	.826
TR	TR2	.822	.088		
TR	TR3	.787	.080		
PU	PU1	.809	—	.587	.810

Latent variable	Item	Standardized loading	*SE*	AVE	CR
PU	PU2	.730	.071		
PU	PU3	.757	.071		
BI	BI1	.772	—	.592	.813
BI	BI2	.804	.081		
BI	BI3	.730	.078		

Note. AVE = average variance extracted; CR = composite reliability; SE = standard error. AVE and CR are shown on the first row of each construct. The loading column reports standardized loadings. SE refers to the corresponding unstandardized loading estimate; the marker indicator fixed for factor scaling has no estimated SE and is shown with an em dash.

Table S15. Confirmatory factor analysis results for the TR–PU combined measurement model (M2)

Latent variable	Item	Standardized loading	*SE*	AVE	CR
AISE	AISE1	.832	—	.637	.875
AISE	AISE2	.816	.057		
AISE	AISE3	.733	.058		
AISE	AISE4	.808	.060		
TR+PU	TR1	.709	—	.394	.788
TR+PU	TR2	.783	.094		
TR+PU	TR3	.741	.086		
TR+PU	PU1	.513	.088		
TR+PU	PU2	.503	.087		
TR+PU	PU3	.431	.086		
BI	BI1	.771	—	.592	.813
BI	BI2	.812	.083		
BI	BI3	.723	.079		

Note. AVE = average variance extracted; CR = composite reliability; SE = standard error. AVE and CR are shown on the first row of each construct. The loading column reports standardized loadings. SE refers to the corresponding unstandardized loading estimate; the marker indicator fixed for factor scaling has no estimated SE and is shown with an em dash.

Table S16. Fornell–Larcker discriminant-validity results for M1

Construct	AISE	TR	PU	BI
AISE	.798			
TR	.316	.783		
PU	.206	.358	.766	
BI	.211	.176	.369	.769

Note. Diagonal entries are square roots of AVE; off-diagonal entries are the reported correlations between constructs. In the finalized analysis, the off-diagonal values reproduce the Pearson construct-score correlations reported in the construct-level correlation analysis.

Table S17. Fornell–Larcker discriminant-validity results for M2

Construct	AISE	TR+PU	BI
AISE	.798		
TR+PU	.317	.628	
BI	.211	.331	.769

Note. Diagonal entries are square roots of AVE; off-diagonal entries are the reported correlations between constructs. In the finalized analysis, the off-diagonal values reproduce the Pearson construct-score correlations reported in the construct-level correlation analysis.

Table S18. HTMT results for M1

Construct	AISE	TR	PU	BI
AISE	—			
TR	.370	—		
PU	.246	.436	—	
BI	.251	.214	.455	—

Note. HTMT = heterotrait–monotrait ratio of correlations; diagonal entries are not applicable.

Table S19. HTMT results for M2

Construct	AISE	TR+PU	BI
AISE	—		
TR+PU	.379	—	
BI	.251	.410	—

Note. HTMT = heterotrait–monotrait ratio of correlations; diagonal entries are not applicable.

S5. Candidate structural models, paths, and full bootstrap results

Tables S20–S28 provide the complete structural-model comparisons, path estimates, bootstrap results, additional tests, and hypothesis-test summary.

Table S20. Full fit indices for the candidate structural models

Model	$\chi^2(df)$	χ^2/df	CFI	TLI	RMSEA (90% CI)	SRMR	Bollen–Stine *p*
A	69.368 (61)	1.137	.996	.994	.020 [.000, .039]	.033	.289
B	123.426 (61)	2.023	.968	.959	.054 [.040, .068]	.085	.001
C	101.915 (60)	1.699	.978	.972	.045 [.029, .059]	.072	.004
C2	67.075 (59)	1.137	.996	.994	.020 [.000, .040]	.027	.284
Extended	67.075 (59)	1.137	.996	.994	.020 [.000, .040]	.027	.284

Note. Model C2 is structurally just-identified and reproduces the global fit of the four-factor CFA; the Extended Model has identical fit. These fit indices are not independent evidence of structural robustness. The identical df and global fit of Model C2 and the Extended Model are reported descriptively; by themselves, they do not establish statistical equivalence and should not be treated as two independent robustness confirmations.

Table S21. Information criteria for the candidate structural models

Model	AIC	BIC	ECVI
A	129.368	245.277	.369
B	183.426	299.334	.523
C	163.915	283.687	.467
C2	131.075	254.711	.373
Extended	131.075	254.711	.373

Note. AIC = Akaike information criterion; BIC = Bayesian information criterion; ECVI = expected cross-validation index. Lower values indicate better relative fit after accounting for model complexity. Values do not establish temporal or causal ordering.

Relative to Model A, ΔAIC/ΔBIC were 54.058/54.057 for Model B, 34.547/38.410 for Model C, and 1.707/9.434 for Model C2 and the Extended Model.

Table S22. Complete path estimates for the five structural models

Model	Path	*B*	β	*SE*	C.R.	*p*	R^2
A	AISE → TR	.329	.378	.055	5.938	< .001	.143
A	TR → PU	.504	.443	.076	6.642	< .001	.196
A	PU → BI	.354	.414	.058	6.094	< .001	.215
A	AISE → BI	.128	.151	.051	2.509	.012	
B	AISE → PU	.278	.278	.063	4.408	< .001	.077
B	PU → TR	.410	.464	.060	6.829	< .001	.216
B	TR → BI	.171	.178	.062	2.738	.006	.081
B	AISE → BI	.168	.199	.053	3.149	.002	
C	AISE → TR	.334	.382	.056	5.975	< .001	.148
C	AISE → PU	.257	.261	.062	4.123	< .001	.068
C	AISE → BI	.120	.141	.058	2.085	.037	.220
C	PU → BI	.353	.410	.060	5.896	< .001	
C	TR → BI	.008	.008	.065	.121	.903	
C2	AISE → TR	.323	.372	.056	5.824	< .001	.138
C2	AISE → PU	.242	.244	.063	3.857	< .001	.059
C2	AISE → BI	.130	.153	.056	2.340	.019	.223
C2	PU → BI	.366	.427	.066	5.574	< .001	
C2	TR → BI	−.030	−.030	.072	−.413	.680	
Extended	AISE → TR	.323	.372	.056	5.824	< .001	.138
Extended	TR → PU	.460	.404	.081	5.657	< .001	.200
Extended	AISE → PU	.093	.094	.064	1.440	.150	
Extended	PU → BI	.366	.427	.066	5.574	< .001	.223
Extended	AISE → BI	.130	.153	.056	2.340	.019	
Extended	TR → BI	−.030	−.030	.072	−.413	.680	

Note. B = unstandardized coefficient; β = standardized coefficient; SE = standard error of B; C.R. = critical ratio. R^2 is reported once for each endogenous construct and represents variance explained in that construct, not an effect of the individual path. For the principal Model C2 direct paths, bias-corrected 95% bootstrap CIs for unstandardized B were [.237, .508] for PU → BI, [−.207, .129] for TR → BI, and [.012, .254] for AISE → BI.

Table S23. Bootstrap summary of model-specific effect estimates

Model	Parameter/path	Estimate	BC lower	BC upper	Percentile lower	Percentile upper	Result
A	AISE → TR → PU → BI indirect effect	.059	.033	.098	.031	.094	Significant
A	AISE → BI direct effect	.128	.023	.239	.019	.236	Significant
A	Total effect	.187	.078	.297	.077	.296	Significant
B	AISE → PU → TR → BI indirect effect	.019	.006	.043	.004	.040	Significant
B	AISE → BI direct effect	.168	.071	.272	.070	.269	Significant

Model	Parameter/path	Estimate	BC lower	BC upper	Percentile lower	Percentile upper	Result
B	Total effect	.187	.092	.290	.090	.289	Significant
C	AISE → TR → BI indirect effect	.003	−.050	.054	−.050	.054	Not significant
C	AISE → PU → BI indirect effect	.091	.044	.157	.042	.153	Significant
C	AISE → BI direct effect	.120	.002	.247	−.004	.243	BC significant / percentile CI includes 0
C	Total effect	.214	.101	.330	.099	.328	Significant
C2	AISE → TR → BI indirect effect	−.010	−.067	.043	−.067	.043	Not significant
C2	AISE → PU → BI indirect effect	.088	.041	.154	.039	.151	Significant
C2	AISE → BI direct effect	.130	.012	.254	.009	.251	Significant
C2	Total effect	.209	.096	.325	.095	.324	Significant
Extended	AISE → TR → BI indirect effect	−.010	−.067	.043	−.067	.043	Not significant
Extended	AISE → PU → BI indirect effect	.034	−.009	.088	−.010	.086	Not significant
Extended	AISE → TR → PU → BI indirect effect	.054	.029	.101	.027	.095	Significant
Extended	AISE → BI direct effect	.130	.012	.254	.009	.251	Significant
Extended	Total effect	.209	.096	.325	.095	.324	Significant

Note. Effect estimates are unstandardized; BC = bias-corrected. All intervals are 95% confidence intervals based on 5,000 bootstrap resamples. In the main manuscript, indirect effects are interpreted as cross-sectional indirect associations.

Table S24. Difference test for the unstandardized TR → BI and PU → BI path coefficients in Model C2

Difference definition	Estimate	*SE*	BC 95% CI lower	BC 95% CI upper	*p*
TR → BI − PU → BI	−.396	.137	−.681	−.142	.001

Note. The difference is defined as TR → BI minus PU → BI. SE = standard error; BC = bias-corrected. The confidence interval is a bias-corrected 95% interval based on 5,000 bootstrap resamples. The reported unstandardized coefficient-difference test is conditional on the latent-variable scaling used in the fitted model; it is not a formal test of equality between the standardized coefficients or a scale-free ranking of the constructs.

Table S25. Two specific indirect associations and their difference in Model C2

Path/difference	Estimate	BC lower	BC upper	Percentile lower	Percentile upper
AISE → TR → BI	−.010	−.067	.043	−.067	.043
AISE → PU → BI	.088	.041	.154	.039	.151
AISE → TR → BI − AISE → PU → BI	−.098	−.200	−.019	−.198	−.018

Note. Estimates are unstandardized; BC = bias-corrected. Both confidence-interval methods use 95% intervals based on 5,000 bootstrap resamples. The difference is defined as the specific indirect association through TR minus that through PU.

Table S26. Disturbance covariance and correlation between eTR and ePU in Model C2

Parameter	*B*	*SE*	C.R.	*p*	*r*	*B* 95% CI lower	*B* 95% CI upper
eTR ↔ ePU	.153	.029	5.192	< .001	.386	.095	.225

Note. B = unstandardized disturbance covariance; SE = standard error of B; C.R. = critical ratio; r = standardized disturbance correlation. The 95% confidence interval refers to B. eTR and ePU denote the disturbance terms of TR and PU, respectively.

Table S27. Chi-square difference tests for nested models

Comparison	$\Delta\chi^2$	Δdf	p	Conclusion
Model A vs Extended Model	2.293	2	.318	Not significant
Model C vs Model C2	34.840	1	< .001	Significant

Note. Model A is nested within the Extended Model; Model C is nested within Model C2. The reported differences are calculated as the more restricted model minus the less restricted model. An ordinary chi-square difference test was not used to compare Model A with Model C2. The Model A restriction test was implemented through the Extended Model parameterization; no ordinary chi-square difference test was used to compare Model A directly with Model C2.

Table S28. Summary of hypothesis tests

Hypothesis	Conclusion
H1: Assessment-context AI self-efficacy is positively associated with trust in GenAI-assisted assessment among design teachers in higher education.	Supported
H2: Assessment-context AI self-efficacy is positively associated with behavioral intention to use GenAI-assisted assessment among design teachers in higher education.	Supported
H3: Trust in GenAI-assisted assessment is positively associated with perceived usefulness among design teachers in higher education.	Supported
H4: Perceived usefulness of GenAI-assisted assessment is positively associated with behavioral intention to use it among design teachers in higher education.	Supported
H5: Within the serial structure specified in Model A, assessment-context AI self-efficacy has a positive specific serial indirect association with behavioral intention through trust and perceived usefulness.	Supported

Note. H1–H4 refer to the prespecified paths in Model A (Table S22); H5 refers to its specific serial indirect association (Table S23). Supported hypotheses do not establish temporal precedence or causation.

S6. Assessment-use-frequency sensitivity analysis (Q14-defined prior-use subset; N = 304)

To examine whether the main findings depended on respondents who reported no prior GenAI-assisted assessment use on the dedicated use-frequency item (Q14), a sensitivity analysis excluded the 48 respondents who selected "Never." For this analysis, prior assessment-related use was operationalized specifically from Q14. The remaining N = 304 respondents selected "Rarely," "Sometimes," "Often," or "Always" and are therefore referred to as the Q14-defined prior-use subset. Q14 was used because it directly assesses assessment-specific use frequency. Q3 served as a broader screening/context-positioning item, and Q16 assessed general GenAI familiarity/exposure; neither was used to define prior-use status in this sensitivity analysis. The label indicates self-reported non-"Never" assessment-use frequency on Q14 only; it does not measure the depth, duration, recency, quality, or proficiency of that experience. The N = 352 sample remains the primary analytical sample because the target outcome is prospective BI and prior assessment-related GenAI use was not an eligibility requirement. Tables S29–S39 report the Q14-defined prior-use sensitivity analyses, including the formal TR → BI versus PU → BI coefficient-difference test.

The four-factor measurement model continued to fit the Q14-defined prior-use subset well, whereas the TR–PU combined model fit substantially worse and showed weak convergent validity for the merged construct. Model A also fit the Q14-defined prior-use subset well ($\chi^2(61) = 68.452$, $\chi^2/df = 1.122$, CFI = .995, TLI = .994, RMSEA = .020, SRMR = .036). Model C2 reproduced the global fit of the four-factor measurement model because its structural part is just-identified; these fit indices are therefore reported descriptively rather than as independent structural evidence. In Model C2, PU → BI remained significant while TR → BI remained nonsignificant, and the formal coefficient-difference test remained significant. Model A retained a significant AISE → TR → PU → BI specific serial indirect association. These results reproduce the main parameter pattern among respondents reporting non-"Never" assessment-related GenAI use on Q14 while directly confirming that the PU → BI coefficient remained significantly larger than the TR → BI coefficient in this Q14-defined subset.

Table S29. Measurement- and structural-model fit in the Q14-defined prior-use subset (N = 304)

Model	χ^2	df	χ^2/df	NFI	IFI	TLI	CFI	GFI	RMSEA	SRMR
Four-factor M1	66.262	59	1.123	.961	.996	.994	.996	.967	.020	.030
Three-factor M2 (TR+PU)	289.578	62	4.671	.830	.861	.824	.860	.844	.110	.086
Model A	68.452	61	1.122	.960	.995	.994	.995	.966	.020	.036
Model C2	66.262	59	1.123	.961	.996	.994	.996	.967	.020	.030

Note. M1 specifies AISE, TR, PU, and BI as separate latent variables; M2 combines TR and PU. Model A is the focal serial structural specification. Model C2 is structurally just-identified and therefore reproduces the global fit of M1; its fit indices are reported descriptively and are not interpreted as independent evidence of structural robustness. The substantially poorer fit of M2 is interpreted together with the item loadings and AVE in Table S31.

Table S30. Confirmatory factor analysis results for four-factor Model M1 in the Q14-defined prior-use subset (N = 304)

Latent variable	Item	Standardized loading	SE	AVE	CR
AISE	AISE1	.817	—	.621	.867
	AISE2	.811	.064		
	AISE3	.713	.064		
	AISE4	.806	.067		
TR	TR1	.752	—	.621	.830
	TR2	.828	.091		
	TR3	.782	.083		
PU	PU1	.802	—	.581	.806
	PU2	.732	.078		
	PU3	.750	.079		
BI	BI1	.760	—	.585	.808
	BI2	.802	.091		
	BI3	.730	.088		

Note. AVE = average variance extracted; CR = composite reliability; SE = standard error. AVE and CR are reported once per construct. The four-factor model retained adequate convergent validity in the Q14-defined prior-use subset. The loading column reports standardized loadings. SE refers to the corresponding unstandardized loading estimate; the marker indicator fixed for factor scaling has no estimated SE and is shown with an em dash.

Table S31. Confirmatory factor analysis results for three-factor Model M2 with TR and PU combined in the Q14-defined prior-use subset (N = 304)

Latent variable	Item	Standardized loading	SE	AVE	CR
AISE	AISE1	.818	—	.621	.867
	AISE2	.810	.064		
	AISE3	.713	.064		
	AISE4	.806	.067		
TR+PU	TR1	.713	—	.411	.801
	TR2	.791	.099		
	TR3	.738	.092		
	PU1	.523	.090		
	PU2	.545	.090		
	PU3	.464	.090		
BI	BI1	.759	—	.585	.808
	BI2	.811	.094		
	BI3	.721	.088		

Note. The merged TR+PU factor had AVE = .411, below .50, and the PU item loadings ranged from .464 to .545. These results, together with the poorer global fit in Table S29, do not support treating TR and PU as one construct in the Q14-defined prior-use

subset. SE values follow the same reporting convention as Table S30: standardized loadings are shown in the loading column, whereas SE corresponds to the estimated unstandardized loading; marker indicators used for factor scaling have no estimated SE.

Table S32. Fornell–Larcker discriminant-validity results for M1 in the Q14-defined prior-use subset (N = 304)

Construct	AISE	TR	PU	BI
AISE	.788			
TR	.293	.788		
PU	.206	.392	.762	
BI	.216	.173	.365	.765

Note. Diagonal entries are square roots of AVE; off-diagonal entries are construct correlations. Each diagonal entry exceeds the corresponding off-diagonal correlations. The off-diagonal entries reproduce the construct-score correlations used in the finalized Q14-defined N = 304 sensitivity analysis.

Table S33. HTMT results for M1 in the Q14-defined prior-use subset (N = 304)

Construct	AISE	TR	PU	BI
AISE	—			
TR	.345	—		
PU	.247	.478	—	
BI	.259	.211	.452	—

Note. HTMT = heterotrait–monotrait ratio of correlations. All values are below .85. These results support discriminant validity among AISE, TR, PU, and BI in the Q14-defined prior-use subset.

Table S34. Fornell–Larcker results for M2 in the Q14-defined prior-use subset (N = 304)

Construct	AISE	TR+PU	BI
AISE	.788		
TR+PU	.300	.641	
BI	.216	.321	.765

Note. These statistics describe the distinction of the merged TR+PU factor from AISE and BI. They do not test whether TR and PU should be combined. That question is evaluated by the poor M2 fit and the weak convergence of the merged factor in Tables S29 and S31. The off-diagonal entries reproduce the construct-score correlations used in the finalized Q14-defined N = 304 sensitivity analysis.

Table S35. HTMT results for M2 in the Q14-defined prior-use subset (N = 304)

Construct	AISE	TR+PU	BI
AISE	—		
TR+PU	.358	—	
BI	.259	.398	—

Note. HTMT values concern the merged TR+PU factor relative to AISE and BI and therefore do not provide evidence that TR and PU themselves are interchangeable.

Table S36. Structural path estimates for Models A and C2 in the Q14-defined prior-use subset (N = 304)

Model	Path	B	β	SE	C.R.	p	R^2
A	AISE → TR	.316	.351	.061	5.145	< .001	.123
A	TR → PU	.517	.481	.077	6.695	< .001	.232
A	PU → BI	.359	.407	.065	5.532	< .001	.213
A	AISE → BI	.136	.159	.056	2.431	.015	
C2	AISE → TR	.310	.345	.062	5.034	< .001	.119
C2	AISE → PU	.236	.244	.066	3.561	< .001	.060
C2	AISE → BI	.141	.165	.060	2.357	.018	.223
C2	PU → BI	.382	.432	.076	5.036	< .001	

Model	Path	B	β	SE	C.R.	p	R^2
C2	TR → BI	−.050	−.052	.077	−.643	.521	

Note. B = unstandardized coefficient; β = standardized coefficient; SE = standard error of B; C.R. = critical ratio. R^2 is reported once for each endogenous construct. Model C2 reproduces the main-sample path pattern: PU → BI is significant whereas TR → BI is not. The formal coefficient-difference test for N = 304 is reported in Table S38.

Table S37. Bootstrap effect estimates for Models A and C2 in the Q14-defined prior-use subset (N = 304)

Model	Parameter/path	Estimate	BC lower	BC upper	Percentile lower	Percentile upper
A	AISE → TR → PU → BI indirect association	.059	.031	.101	.029	.098
A	AISE → BI direct effect	.136	.014	.256	.016	.257
A	Total effect	.194	.072	.311	.074	.314
C2	AISE → TR → BI specific indirect association	−.015	−.083	.039	−.083	.040
C2	AISE → PU → BI specific indirect association	.090	.040	.161	.039	.159
C2	AISE → BI direct effect	.141	.007	.272	.010	.275
C2	Total effect	.216	.088	.340	.091	.344

Note. Effect estimates are unstandardized; BC = bias-corrected. All intervals are 95% confidence intervals based on 5,000 bootstrap resamples. Indirect effects are interpreted as cross-sectional indirect associations. The serial association in Model A and the specific association through PU in Model C2 are significant because their intervals exclude zero; the specific association through TR in Model C2 is not significant.

Table S38. Difference test for the unstandardized TR → BI and PU → BI path coefficients in the Q14-defined prior-use subset (N = 304)

Difference definition	Estimate	SE	BC 95% CI lower	BC 95% CI upper	p
TR → BI − PU → BI	−.432	.159	−.777	−.148	.003

Note. The difference is defined as TR → BI minus PU → BI. SE = standard error; BC = bias-corrected. The 95% confidence interval is based on 5,000 bootstrap resamples. The reported unstandardized coefficient-difference test is conditional on the latent-variable scaling used in the fitted model; it is not a formal test of equality between the standardized coefficients or a scale-free ranking of the constructs.

Table S39. Two specific indirect associations and their difference in Model C2 in the Q14-defined prior-use subset (N = 304)

Path/difference	Estimate	BC lower	BC upper	Percentile lower	Percentile upper
AISE → TR → BI	−.015	−.083	.039	−.083	.040
AISE → PU → BI	.090	.040	.161	.039	.159
AISE → TR → BI − AISE → PU → BI	−.106	−.218	−.023	−.216	−.022

Note. Estimates are unstandardized; BC = bias-corrected. Both interval methods use 95% intervals based on 5,000 bootstrap resamples. The difference is defined as the specific indirect association through TR minus that through PU.

S7. Additional sensitivity analyses

S7.1. Assessment-use-frequency covariate sensitivity analysis (Model C2 + UseExp)

As an additional sensitivity analysis, the original five-category GenAI-assisted assessment use-frequency variable (Q14) was entered as an observed covariate in Model C2. UseExp was specified as a predictor of TR, PU, and BI and was allowed to covary with AISE; the eTR ↔ ePU disturbance covariance remained freely estimated as in Model C2. This analysis was used to examine whether the main PU–BI/TR–BI parameter pattern changed after accounting for reported use frequency. It is not interpreted causally. Tables S40–S43 report the full results.

Table S40. Fit indices for Model C2 + UseExp (N = 352)

Model	χ^2	df	χ^2/df	NFI	IFI	TLI	CFI	GFI	RMSEA	SRMR
Model C2 + UseExp	79.957	68	1.176	.960	.994	.992	.994	.967	.022	.028

Note. UseExp = self-reported frequency of GenAI-assisted assessment use (Q14), coded in ascending order from "Never" to "Always." It was modeled as a single ordered score, so this covariate analysis assumes equal score increments and linear associations rather than estimating separate effects for all five response categories.

Table S41. Path estimates for Model C2 + UseExp (N = 352)

Model	Path/parameter	B	β/r	SE	C.R.	p	R^2
C2 + UseExp	AISE → TR	.330	.380	.056	5.931	< .001	.146
C2 + UseExp	UseExp → TR	−.052	−.088	.033	−1.576	.115	
C2 + UseExp	AISE → PU	.239	.240	.063	3.797	< .001	.061
C2 + UseExp	UseExp → PU	.024	.036	.039	.617	.537	
C2 + UseExp	AISE → BI	.135	.159	.056	2.419	.016	.226
C2 + UseExp	UseExp → BI	−.032	−.055	.032	−.994	.320	
C2 + UseExp	PU → BI	.368	.431	.066	5.610	< .001	
C2 + UseExp	TR → BI	−.037	−.038	.073	−.510	.610	
C2 + UseExp	AISE ↔ UseExp	.068	.090	.043	1.575	.115	
C2 + UseExp	eTR ↔ ePU	.155	.392	.030	5.253	< .001	

Note. B = unstandardized coefficient or covariance; β/r = standardized path coefficient for directed paths and standardized correlation for covariance rows; SE = standard error of B; C.R. = critical ratio. R^2 is reported once for each endogenous construct. eTR and ePU denote the disturbance terms of TR and PU, respectively.

Table S42. Formal path-difference test and covariate bootstrap results for Model C2 + UseExp (N = 352)

Parameter	Estimate	SE	BC 95% CI lower	BC 95% CI upper	p
TR → BI − PU → BI	−.405	.138	−.688	−.145	< .001
UseExp → TR	−.052	.033	−.120	.014	.117
UseExp → PU	.024	.039	−.058	.107	.569

Parameter	Estimate	SE	BC 95% CI lower	BC 95% CI upper	p
UseExp → BI	−.032	.032	−.098	.032	.324

Note. BC = bias-corrected. Confidence intervals are 95% intervals based on 5,000 bootstrap resamples. The bootstrap p values for UseExp paths are reported separately from the conventional AMOS Regression Weights p values shown in Table S41. The reported unstandardized coefficient-difference test is conditional on the latent-variable scaling used in the fitted model; it is not a formal test of equality between the standardized coefficients or a scale-free ranking of the constructs.

Table S43. Two specific indirect associations and their difference in Model C2 + UseExp (N = 352)

Path/difference	Estimate	BC lower	BC upper	Percentile lower	Percentile upper
AISE → TR → BI	−.012	−.072	.042	−.072	.042
AISE → PU → BI	.088	.041	.154	.039	.150
AISE → TR → BI − AISE → PU → BI	−.100	−.204	−.022	−.201	−.020

Note. Estimates are unstandardized; BC = bias-corrected. Both interval methods use 95% intervals based on 5,000 bootstrap resamples.

S7.2. Constrained ULMF common-method sensitivity analysis

Because AISE, TR, PU, and BI were measured in the same self-report questionnaire, a constrained CFA-based ULMF model was estimated as a stronger post-hoc sensitivity check, consistent with general latent-method-factor approaches to common-method variance (Podsakoff et al., 2003; Richardson et al., 2009). All 13 observed indicators retained their substantive-factor loadings and additionally loaded on an orthogonal common method factor. The unstandardized method-factor loadings were constrained to equality across items, and the method factor was scaled for identification. The model converged normally, with no negative error variances, standardized estimates greater than 1, or other improper estimates. The analysis is interpreted as a sensitivity check rather than as evidence that common-method bias has been eliminated.

Table S44. Fit comparison of the four-factor measurement model (M1) and the constrained ULMF model (N = 352)

Model	χ^2	df	χ^2/df	NFI	IFI	TLI	CFI	GFI	RMSEA	SRMR
Four-factor M1	67.075	59	1.137	.967	.996	.994	.996	.971	.020	.027
Constrained ULMF	67.064	58	1.156	.967	.995	.994	.995	.971	.021	.027

Note. The constrained ULMF model is a post-hoc common-method sensitivity analysis. Its fit is compared descriptively with M1; the analysis does not rule out common-method variance.

Table S45. Substantive standardized loadings, AVE, and CR in the four-factor measurement model (M1) and the constrained ULMF model (N = 352)

Construct	Item	M1 loading	ULMF loading	M1 AVE	ULMF AVE	M1 CR	ULMF CR
AISE	AISE1	.831	.820	.637	.615	.875	.864
AISE	AISE2	.816	.801				
AISE	AISE3	.733	.717				
AISE	AISE4	.808	.795				
TR	TR1	.739	.723	.614	.591	.826	.812
TR	TR2	.822	.809				
TR	TR3	.787	.772				

Construct	Item	M1 loading	ULMF loading	M1 AVE	ULMF AVE	M1 CR	ULMF CR
PU	PU1	.809	.796	.587	.566	.810	.796
PU	PU2	.730	.715				
PU	PU3	.757	.743				
BI	BI1	.772	.757	.592	.567	.813	.797
BI	BI2	.804	.788				
BI	BI3	.730	.713				

Note. AVE = average variance extracted; CR = composite reliability. Relative to M1, substantive standardized loadings changed by approximately .011–.017, AVE by .021–.025, and CR by .011–.016. All indices remained within acceptable ranges.

S8. Standardized result diagrams for alternative and extended models

Model A and Model C2 are the core structural results, and their standardized diagrams are presented in Figures 2 and 3 of the main manuscript. Figures S2–S4 present Model B, Model C, and the Extended Model to document the reverse-direction, strict independent-parallel, and extended structural specifications. These diagrams do not provide additional evidence about temporal or causal ordering.

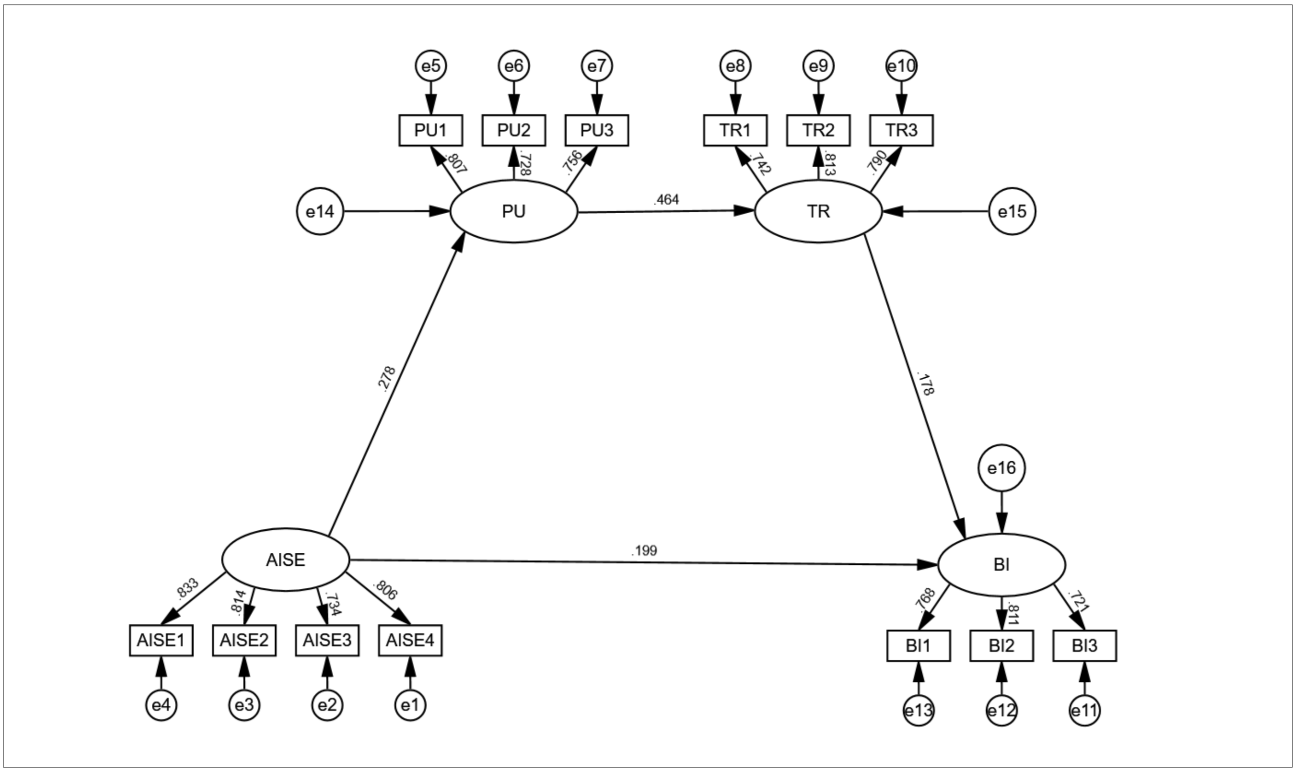

Figure S2. Standardized results for Model B

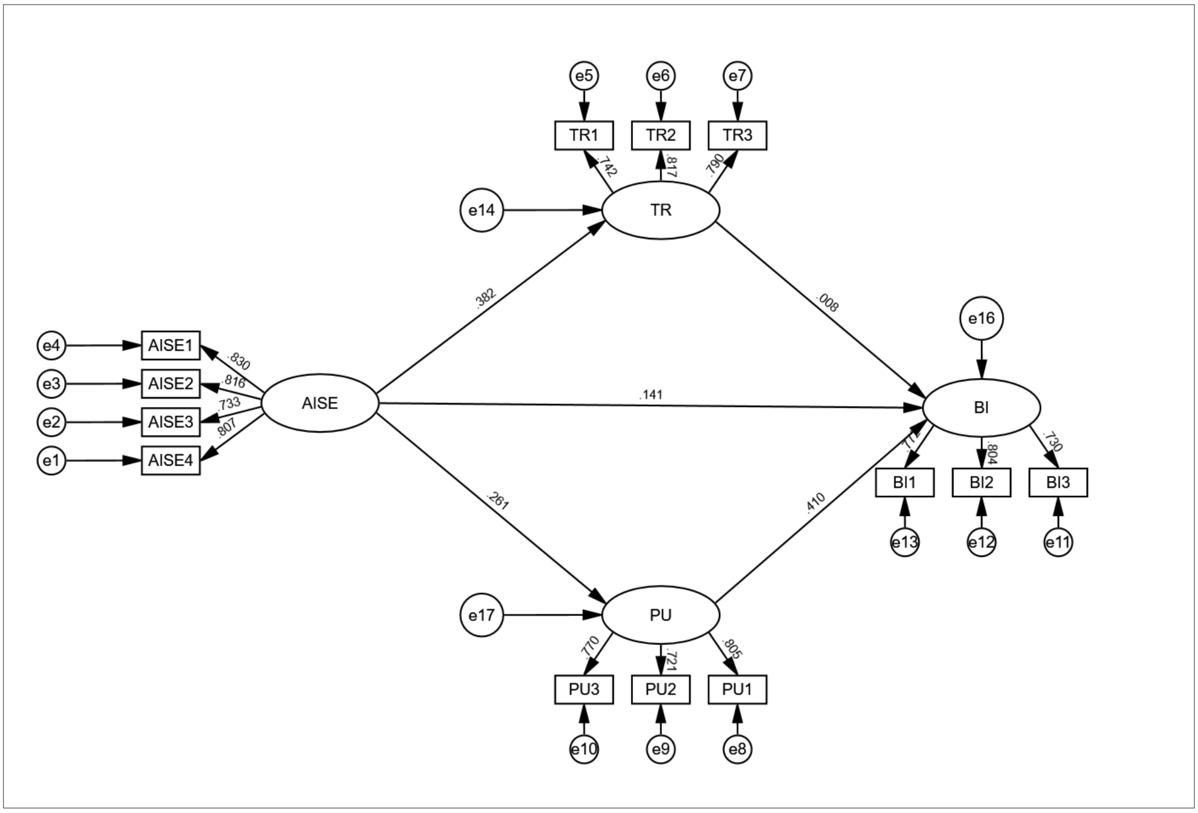

Figure S3. Standardized results for Model C

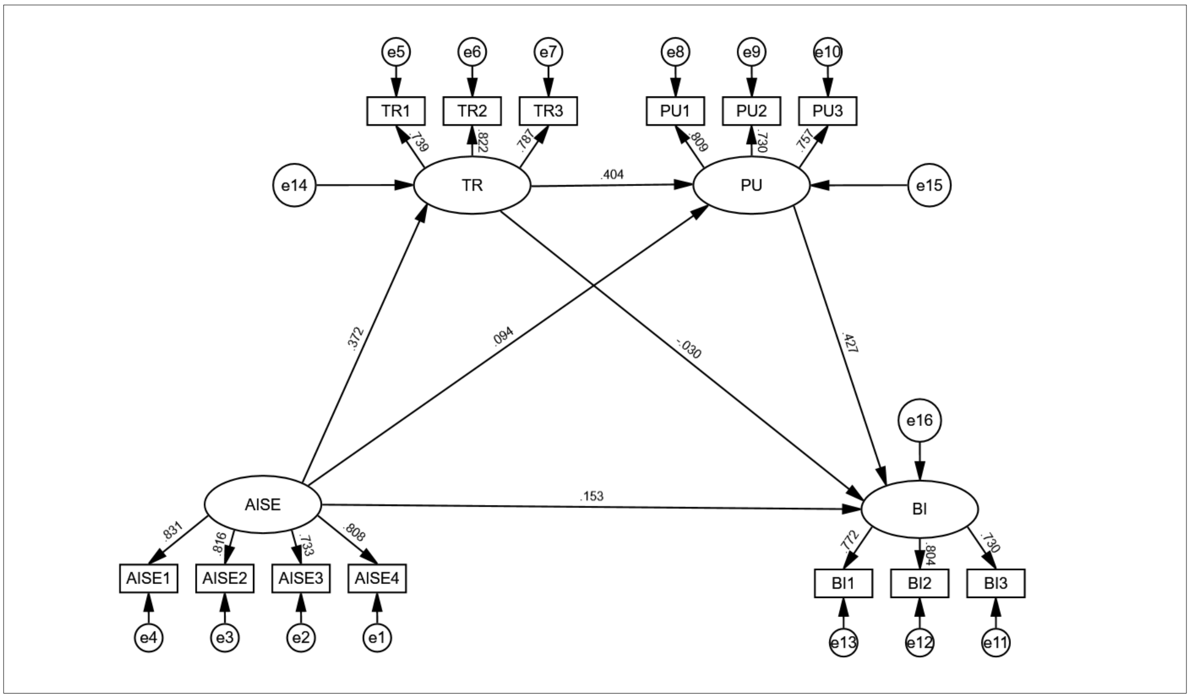

Figure S4. Standardized results for the Extended Model

Note. Coefficients in Figures S2–S4 are standardized estimates; the circles labeled e1, e2, etc. denote measurement errors or structural disturbances. Complete path coefficients and p values are reported in Table S22. Standardized diagrams for Model A and Model C2 are provided in Figures 2 and 3 of the main manuscript; Figures S2–S4 document Model B, Model C, and the Extended Model, respectively.

References

Davis, F. D. (1989). Perceived usefulness, perceived ease of use, and user acceptance of information technology. *MIS Quarterly, 13*(3), 319–340. https://doi.org/10.2307/249008

Jian, J. Y., Bisantz, A. M., & Drury, C. G. (2000). Foundations for an empirically determined scale of trust in automated systems. *International Journal of Cognitive Ergonomics, 4*(1), 53–71. https://doi.org/10.1207/S15327566IJCE0401_04

McGrath, M. J., Lack, O., Tisch, J., & Duenser, A. (2025). Measuring trust in artificial intelligence: Validation of an established scale and its short form. *Frontiers in Artificial Intelligence, 8*, 1582880. https://doi.org/10.3389/frai.2025.1582880

Podsakoff, P. M., MacKenzie, S. B., Lee, J.-Y., & Podsakoff, N. P. (2003). Common method biases in behavioral research: A critical review of the literature and recommended remedies. Journal of Applied Psychology, 88(5), 879–903. https://doi.org/10.1037/0021-9010.88.5.879

Richardson, H. A., Simmering, M. J., & Sturman, M. C. (2009). A tale of three perspectives: Examining post hoc statistical techniques for detection and correction of common method variance. Organizational Research Methods, 12(4), 762–800. https://doi.org/10.1177/1094428109332834

Venkatesh, V., Morris, M. G., Davis, G. *B*., & Davis, F. D. (2003). User acceptance of information technology: Toward a unified view. *MIS Quarterly, 27*(3), 425–478. https://doi.org/10.2307/30036540

Xia, Q., Yang, Y., Weng, X., Cilsalar-Sagnak, H., Cheng, W. K., & Chiu, T. K. F. (2026). Generative artificial intelligence competency self-efficacy scale for teachers and researchers (GAICS-TR) in higher education. *Journal of Computers in Education, 13*(2), 769–793. https://doi.org/10.1007/s40692-025-00373-y
